# Gene autoregulation via intronic microRNAs and its functions

**DOI:** 10.1186/1752-0509-6-131

**Published:** 2012-10-10

**Authors:** Carla Bosia, Matteo Osella, Mariama El Baroudi, Davide Corà, Michele Caselle

**Affiliations:** 1 Human Genetics Foudation (HuGeF), V. Nizza 52, Torino, I-10126, Italy; 2 Center for Complex Systems in Molecular Biology and Medicine, University of Torino, V. Accademia Albertina 13, I-10100 Torino, Italy; 3 Genomic Physics Group, UMR 7238 CNRS “Microorganism Genomics”, Paris, 75006, France; 4 Université Pierre et Marie Curie, Paris, 75006, 15 rue de L’École de Médecine, France; 5 National Research Council (CNR), Institute of Informatics and Telematics (IIT) and Institute of Clinical Physiology (IFC), Laboratory for Integrative System Medicine (LISM), Via Giuseppe Moruzzi 1, Pisa, I-56124, Italy; 6 IRC@C: Institute for Cancer Research at Candiolo, School of Medicine, University of Torino, Str. Prov. 142, Km. 3.95, Torino, Candiolo I-10060, Italy; 7 Dipartimento di Fisica Teorica and INFN, University of Torino, V. Pietro Giuria 1Torino, I-10125, Italy

## Abstract

**Background:**

MicroRNAs, post-transcriptional repressors of gene expression, play a pivotal role in gene regulatory networks. They are involved in core cellular processes and their dysregulation is associated to a broad range of human diseases. This paper focus on a minimal microRNA-mediated regulatory circuit, in which a protein-coding gene (host gene) is targeted by a microRNA located inside one of its introns.

**Results:**

Autoregulation via intronic microRNAs is widespread in the human regulatory network, as confirmed by our bioinformatic analysis, and can perform several regulatory tasks despite its simple topology. Our analysis, based on analytical calculations and simulations, indicates that this circuitry alters the dynamics of the host gene expression, can induce complex responses implementing adaptation and Weber’s law, and efficiently filters fluctuations propagating from the upstream network to the host gene. A fine-tuning of the circuit parameters can optimize each of these functions. Interestingly, they are all related to gene expression homeostasis, in agreement with the increasing evidence suggesting a role of microRNA regulation in conferring robustness to biological processes. In addition to model analysis, we present a list of bioinformatically predicted candidate circuits in human for future experimental tests.

**Conclusions:**

The results presented here suggest a potentially relevant functional role for negative self-regulation via intronic microRNAs, in particular as a homeostatic control mechanism of gene expression. Moreover, the map of circuit functions in terms of experimentally measurable parameters, resulting from our analysis, can be a useful guideline for possible applications in synthetic biology.

## Background

microRNAs (miRNAs) are small (about 22 nucleotides) single-strand RNAs able to interfere post-transcriptionally with the protein production of their targets. Targeting a vast proportion of protein-coding genes [1-3], miRNA-mediated regulation composes an important layer in gene regulatory networks. The implication of miRNAs in several core cellular processes [4-7] as well as in many human diseases [8,9] further confirms their biological importance.

Approximately half of the miRNA genes can be found in intergenic regions (between genes), whereas the intragenic miRNAs (inside genes) are predominantly located inside introns and usually oriented on the same DNA strand of the host gene [10] (a trend further confirmed by our bioinformatic analysis shown in a following section). Intergenic miRNA genes present their own promoter region [11,12] and their expression is expected to be regulated by the same molecular mechanisms that control the expression of protein-coding genes. On the other hand, experimental and computational results are consistent with the idea that same-strand intronic miRNAs are co-transcribed with their host gene [13-17], and then processed to finally become mature functional miRNAs [18,19] (although exceptions to this common scheme of co-transcripton have been reported [20-22]).

The host-miRNA co-expression can have a specific functional role. In fact, an intronic miRNA can support the function of its host gene by silencing genes that are functionally antagonistic to the host [23], or more generally act synergistically with the host by coordinating the expression of genes with related functions [24].

In addition to this “cooperative” miRNA-host relation, different studies showed that intronic miRNAs can directly regulate the expression of their host gene, establishing a negative feedback regulation [10,25,26]. In particular, instances of negative autoregulatory feedbacks via intronic miRNAs were firstly found by expression analysis in human [25]. More recently, two independent large-scale bioinformatic analysis, based on different algorithms of target prediction, claimed that the occurency of intronic miRNA-mediated self-loops (iMSLs) in the human regulatory network is significantly higher than expected by chance [10,26]. The over-representation of such regulatory module can be interpreted as a sign of evolutionary positive selection that has led to an accumulation of a specific topology able to perform useful elementary regulatory tasks [27]. In addition, two iMSL circuits have been confirmed experimentally: regulation of EGFL7 by its intronic miRNA miR-126 [28,29] and regulation of ARPP-21 by miR-128b [26]. Both regulations were associated to relevant biological functions, the former playing a role in cancer proliferation [28], while the latter in vertebrate brain physiology [26].

The combination of all these pieces of evidence suggests that iMSLs are an often exploited and presumably functionally relevant regulatory circuitry. The open question concerns the peculiar functions that an iMSL can accomplish and that could have thus driven their pervasive spreading in the human regulatory network. Moreover, it would be interesting to understand what specificities of post-transcriptional autoregulation by miRNAs can make them better suited to fullfil certain tasks with respect to the trascriptional self-regulation, so widely used in bacteria [30]. In this paper we address these questions by modeling the dynamical and stochastic behaviour of the iMSL circuit and comparing its properties to those of alternative regulatory strategies such as constitutive expression and transcriptional self repression.

Our results show that, despite of its minimal topology, the iMSL circuitry can implement different biological functions. It can speed-up the host gene protein production in response to an activating signal, while delaying its switching-off kinetics when the activation drops; it can buffer fluctuations coming from the upstream network, and generate complex behaviours like a host gene expression response obeying “Weber’s law” (i.e. the magnitude of the response depends only on the fold change of the input signal). While these different functions can be optimized individually, by tuning parameters like molecular production/degradation rates, it will be shown that they all represent different ways of making the host gene expression robust to external fluctuations. Therefore, autoregulation via intronic miRNAs can generally represent an efficient homeostatic control of the host gene expression, in agreement with the observation that miRNAs are often involved in signaling networks to ensure homeostasis and gene expression robustness [31-34]. In addition to model analysis, we present our own bioinformatical search for iMSLs in human to further assess their statistical over-representation and to propose the best predicted candidates for possible future experimental tests.

Besides the understanding of the role of endogenous iMSLs, our results can be useful for the growing field of synthetic biology [35,36], which has succesfully started to make use of RNA-based post-transcriptional regulations [37,38]. The function-topology map presented in this paper can contribute to draw up the manual of biological circuits that carry out specific functions for synthetic engineering, adding a simple and efficient wiring strategy that can increase systems’ robustness in different conditions. A synthetic realization of an iMSL has been indeed recently produced and proven to be effective in reducing the expression dependency on gene dosage [39]. Therefore, the potential additional functions we will show associated to iMSLs could be tested in the near future.

## Results and discussion

### Outline of the model

**Figure 1:**
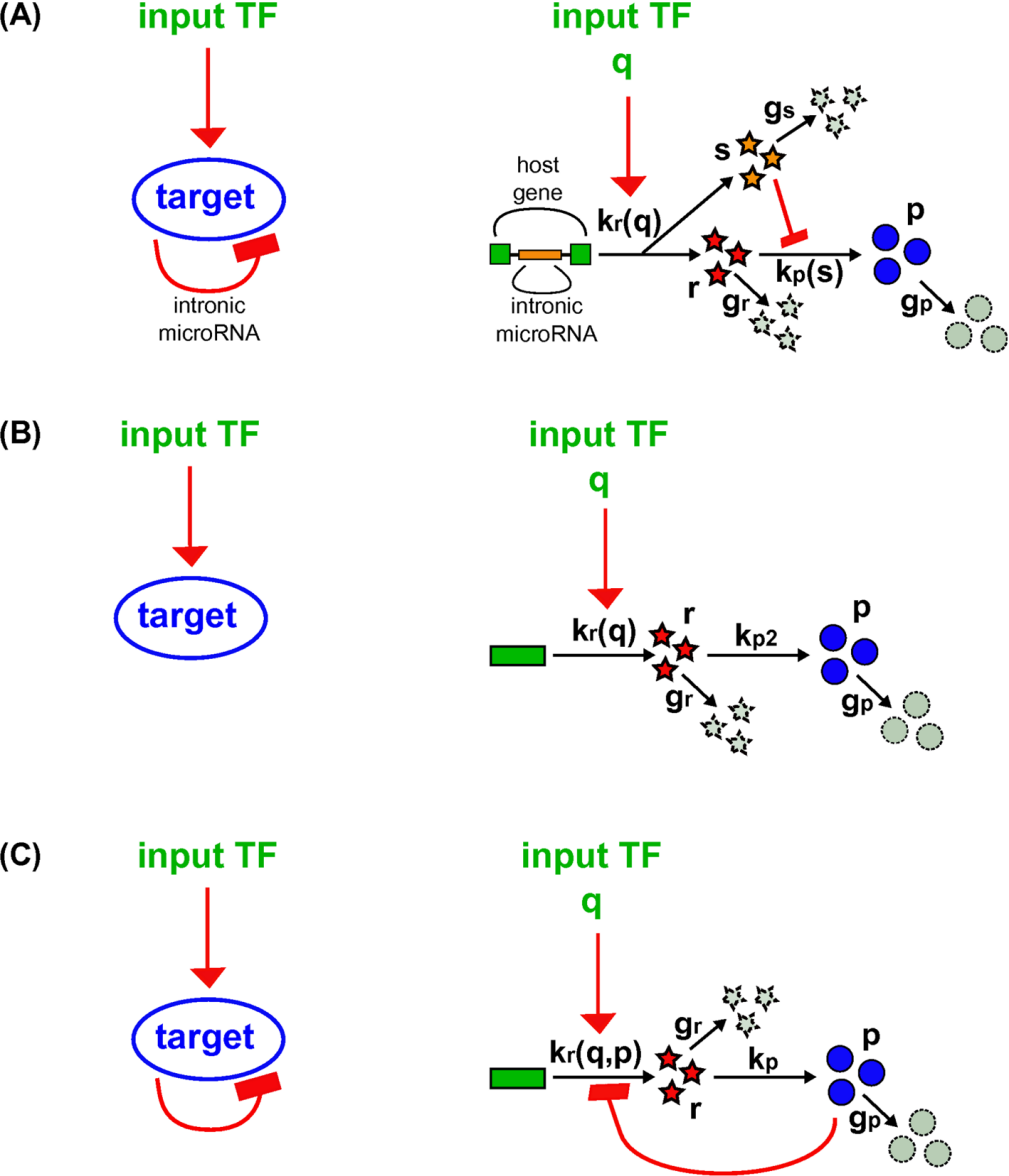
**Representation of iMSL and the two circuits used for comparison.** Schematic views of **(A)** an intronic miRNA-mediated self-loop (iMSL); **(B)** a gene simply activated by a TF (sTF); **(C)** a transcriptional self-regulation (tSL). A more detailed representation of the three circuits is on the right of the figure. Green rectangles are DNA-genes; *s* and *r* are the transcribed miRNAs and mRNAs (orange and red stars respectively) which can eventually be degraded (broken grey stars). mRNAs can be translated into proteins *p* (blue circles) and proteins can be degraded (broken grey circles). The reaction rates are reported along the corresponding black arrows: *k*_*r*_(*q*) and *k*_*r*_(*q*,*p*) for transcription; *k*_*p*_(*s*), *k*_*p*2_ and *k*_*p *_for translation; *g*_*s*_, *g*_*r *_and *g*_*p *_for degradation. Red arrows represent activations, while red lines ending in bars are repressions. For the iMSL and the sTF, the transcription rates are functions of the amount of TFs *q*, while for the tSL the transcription rate is also a function of the target protein *p*. In the iMSL, miRNA regulation makes the rate of translation a function of the amount of miRNAs *s*.

We are interested in a model of iMSLs that can capture the fundamental properties of the circuit, but simplified enough to avoid the introduction of too many free parameters that would make an exploration of the parameter space unfeasible. In this view, the essential steps of transcription, translation, degradation and interactions between genes are taken into account as summarized in Figure 1A. The host gene is assumed to be under the control of an activating transcription factor (TF) with concentration *q*, in order to study the dynamical and stochastic properties of the circuit in presence of upstream input signals. The activation is modeled, as usual in this type of descriptions [30,40], representing the transcription rate *k*_*r*_(*q*) of the target as a Michaelis-Menten function of TF concentration *q*:

(1) $$k_{r}(q)=\frac{k_{r}q}{h_{r}+q},$$

where *k*_*r *_represents the maximum transcription rate in the fully activated state, while *h*_*r*_ is a dissociation constant specifying the TF concentration at which the transcription rate is half of its maximum value. However, the analysis can be straightforwardly extended to the case of a Hill function (substituting *q* with *q*^*n*^ and *h*_*r*_ with $h_{r}^{n}$), if in presence of cooperativity.

On the other hand, miRNAs can exert their action repressing translation or inducing degradation of their target mRNAs [41]. We construct our model supposing an action on target translation. While most of the results shown in this paper are independent of this choice, some dynamical properties of the circuit can actually change if miRNA action is mainly due to induction of mRNA degradation. This issue is discussed in more detail in Additional file 1.

A phenomenological description based on nonlinear functions has been proven to be effective in modeling RNA interference in mammals [42], and was previously applied in computational analysis [43,44]. Along these lines, we assume that miRNA regulation makes the target translation rate *k*_*p*_(*s*) a repressive Michaelis-Menten-like function of the number of miRNAs (*s*):

(2) $$k_{p}(s)=\frac{k_{p}}{1+\frac{s}{h}}.$$

The dissociation constant *h* establishes the miRNA level at which the target translation rate is half of its maximum value *k*_*p*_. With the regulatory interactions defined in Equations 1 and 2, it is possible to represent the dynamics of the circuit in Figure 1A by a set of differential equations:

(3) $$\begin{array}{l} \frac{\text{dr}}{\text{dt}}=k_{r}(q)-g_{r}r\\ \frac{\text{ds}}{\text{dt}}=k_{r}(q)-g_{s}s\\ \frac{\text{dp}}{\text{dt}}=k_{p}(s)\;r-g_{p}p,\; \end{array}$$

where *r* and *p* are the levels of host gene mRNA and protein products, *s* is the level of miRNAs, and *g*_*i*_represents the degradation rate of the molecular species *i*. As discussed in the introduction, intronic miRNAs (same-strand with the host) are expected to be co-transcribed with their host gene, therefore their production rate *k*_*r*_(*q*) has the same dependence on the input TF level.

A different representation was introduced in the context of bacterial sRNA regulation [45-47] and subsequently applied with slight modifications to eukaryotic miRNA regulation [48]. In this representation, the degree of catalicity, i.e. the ability of a miRNA to affect multiple mRNAs without being degraded, was parametrized explicitly [45]. The use of an effective phenomenological function (like the one in Equation 2) implicitly assumes a catalytic action, as commonly believed for miRNAs [42].

Moreover, miRNA biogenesis is a complex and highly regulated multi-step process (see [5,18,49] for a review) that finally leads to a mature miRNA loaded into the RNA-induced silencing complex (RISC) which is actually the active form that can downregulate the target mRNAs. Recent models of regulation by small RNAs have tried to build a more detailed modeling framework taking into account these processing steps [39,50], for example including miRNA incorporation into the RISC complex as a reversible binding reaction. Such detailed modeling approaches have succesfully explained results from synthetic biology experiments. However, a more comprehensive mathematical representation of the biological processes involved comes at the expense of an increased number of free parameters, making more difficult an extensive exploration of the parameter space. While in our modeling strategy the dynamics of miRNA biogenesis is not included, in the supporting information (Additional file 1) we report the analysis of the possible effects of the delay that the biogenesis process can introduce between miRNA transcription and miRNA-mediated repression. More generally, the relations between different possible models of miRNA regulation are discussed in detail in the supporting information (Additional file 1), where it is shown that most of the results that will be presented in the following are essentially independent on the modeling strategy, provided that certain generic conditions on the parameters are satisfied.

In an analogous manner, it is possible to model the two circuits that we will use for comparison: a gene simply activated by the TF (sTF) without any feedback regulation (scheme in Figure 1B) and a transcriptional self-loop (tSL), in which the negative feedback is realized through transcriptional repression (scheme in Figure 1C). The properties of each circuit will be compared using a so called *mathematically controlled comparison*[30]: all the common parameters will be kept to equivalent values, constraining the remainders so as to achieve the same steady state of protein concentration.

A deterministic description based on ordinary differential equations can effectively describe the mean kinetic behaviour of genetic circuits, thus its predictions can be tested with experiments based on averages over cell populations. In fact, equivalent mathematical treatments have correctly predicted the dynamical features of several endogenous and synthetics circuits [27,30]. However, since gene expression is inherently a stochastic process [51-53], we will also make use of a stochastic description based on a master equation approach, that has Equations 3 as a “mean-field” limit (complete model in Additional file 1). To compare the stochastic properties and the noise susceptibility of the three regulatory strategies in Figure 1, we calculated analytically the relative fluctuations in protein level *p* at steady state and confirmed our results with Gillespie simulations (see the Methods section for details on simulations).

Autoregulation via intronic miRNAs has many of the structural properties of miRNA-mediated incoherent feedforward loops, that represent a diffused and functionally relevant motif in regulatory networks [25,44,54-56]. In fact, iMSLs can be thought as a mimimal feedforwad topology with perfect co-expression of the target gene and the miRNA buffering node, and thus, as previously observed [39], can be considered a special case of post-transcriptional incoherent feedforward loops. As a matter of fact, many of the functions that we will show the iMSL can perform are consistent with the feedforward nature of this circuit, and the analysis presented in this paper could be easily generalized to miRNA-mediated incoherent feedforward loops, adding new pieces to our understanding of microRNA regulation in simple circuits.

### Response times to external signals are altered by autoregulation via intronic microRNAs

The response of a transcriptional unit to a stimulus, such as a change in a TF concentration, is steered by the lifetime of its mRNA and protein products. A fast protein turnover speeds up the kinetics, but with a consequent high metabolic cost, while in the case of long-living proteins the timescale of changes in concentration can be comparable to the cell cycle time [30,57], which can be of several hours. However, the dynamics of a gene expression also depends strongly on the regulatory circuitry in which the gene is embedded. For example, it has been proven that negative transcriptional self regulation (like the one in Figure 1C) and incoherent feedforward loops speed up the expression rise-time after induction [57,58], while coherent feedforwad loops introduce delays [59].

We address in this section the question of how the host gene kinetics is changed by being a target of its intronic miRNA. To this aim, we consider two opposite simplified situations: (i) a sudden activating signal that fully saturates the promoter, and (ii) the opposite case of an istantaneous drop of the activating signal that completely switches off transcription. Case (i) can be studied assuming that at *t*=0 the transcription rate *k*_*r*_(*q*) switches from zero to its maximum value *k*_*r*_, and measuring the response time *T*_*ON*_defined as the time needed to reach half of the final protein steady state. In other words, we integrate numerically Equations 3 to calculate the time *T*_*ON *_such that *p*(*T*_*ON*_)/*p*_*ss *_= 0.5 (where *p*_*ss*_ is the final steady-state protein level), starting from the condition *r*(0) =* s*(0) =* p*(0) = 0. In case (ii), in which we assume a drop of the activating signal at *t *= 0, we can similarly define a response time *T*_*OFF*_ looking at the decrease of *p*(*t*) after a switch of the transcription rate from *k*_*r *_to zero at time *t *= 0. The same analysis is performed on a sTF (scheme in Figure 1B) and a tSL (scheme in Figure 1C) for comparison. The response time *T*_0_ of the simple transcription unit sTF is used as a normalization, since *T*_*ON*(*OFF*)_/*T*_0_ is a measure of how much a circuit can alter the response time with respect to an unregulated gene.

**Figure 2:**
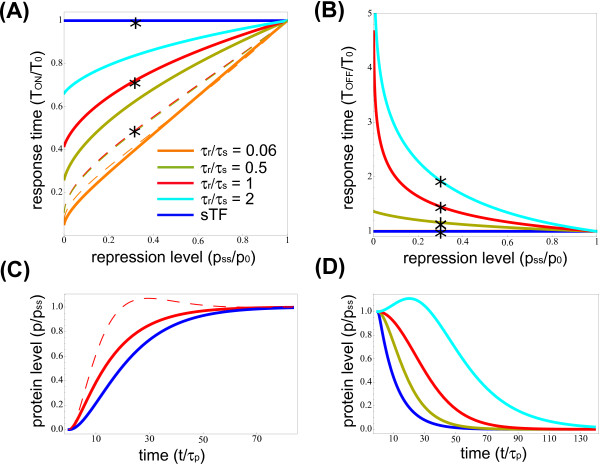
**Autoregulation via intronic miRNAs speeds up the host gene activation and delays its deactivation.(A)** Activation response time: response time *T*_*ON*_, normalized by sTF response time *T*_0_, plotted as a function of repression level *p*_*ss*_/*p*_0_(*p*_*ss*_/*p*_0_ = 1 means no repression) for different values of mRNA/miRNA lifetimes. Both iMSL and tSL (continuous and dashed lines) accelerate the response time with respect to sTF (blue line). Colors correspond to values of mRNA and miRNA relative stability (*τ*_*r*_/*τ*_*s*_), achieved keeping mRNA and protein degradation rates (*g*_*r*_, *g*_*p*_) fixed while varying miRNA degradation rate (*g*_*s*_). The half-life ratio *τ*_*r*_/*τ*_*s *_affects also the tSL dynamics, but the dependence is weak (the corresponding curves tend to collapse). **(B)** Deactivation response times: response time *T*_*OFF*_, normalized by *T*_0_, is shown for different repression levels. The blue line corresponds to the sTF. The response time for iMSL is plotted for different miRNA half-lives (same color code of A). The iMSL induces a delayed host gene response in the same repression range that consents a *T*_*ON*_reduction. **(C)** Example of target protein temporal evolution in activation for the three circuitries. The parameter values correspond to stars in plot A. Time is in protein half-life units. **(D)** Example of iMSL target protein dynamics in deactivation for different mRNA/miRNA half-lives (stars in plot B). The parameter setting for this panel is the following: protein half-life *τ*_*p *_= 8 hours, mRNA half-life *τ*_*r *_= 30 minutes, *h *= 1000, *k*_*r *_= 0.212819*s*^−1^, *k*_*p *_= 0.0048*s*^−1^.

Many previous analyses of genetic circuit dynamics have assumed short-living mRNAs with respect to proteins [57-59]. Within this assumption, the mRNA dynamics can be neglected since the timescales are governed by the protein kinetics. While this is usually a safe approximation in bacteria, in eukaryotes the phenomenology can be more complex. In mammals, the mRNA half-life can range from minutes to about 24 hours [60,61], with typical values in the range of 5−10 hours [62,63]. Similarly, protein lifetimes cover quite a wide range, from minutes to several days [64]. MiRNAs are usually stable molecules with an half-life that can span days [65,66], but there are cases of short-living miRNAs, as many miRNAs expressed in human brain [67]. Moreover, the miRNA turnover seems widely regulated as it happens for mRNAs and proteins [68]. In summary, while the situation in which proteins are more stable than the corresponding transcripts could still be frequent, a variety of specific cases is expected. Therefore, we decided to take into account the mRNA dynamics and explore different regimes of molecules’ half-lives. Indeed, we will show that the dynamical response of the iMSL circuit depends crucially on the ratio between mRNA and miRNA half-lives (*τ*_*r*_/*τs*). In Figure 2A, the normalized response time *T*_*ON*_/*T*_0_ to activation is plotted as a function of the repression level measured as *p*_*ss*_/*p*_0_, where *p*_*ss*_ is the final steady-state protein concentration, while *p*_0_ is the steady-state protein concentration in absence of negative regulation. The response time of the iMSL (continuous lines) and the tSL (dashed lines) is reported for different values of the half-life ratio *τ*_*r*_/*τ*_*s*_. As a first result, the iMSL can speed up the response time with a comparable efficiency with respect to its transcriptional counterpart, especially when mRNAs are degraded fastly enough. On the other hand, when miRNAs are short-living with respect to mRNAs, they will reach their final concentration faster than mRNAs, thus blocking more quickly the initial rise in target protein concentration. Therefore, the timescales of mRNA and miRNA dynamics, determined by their half-lives, define the circuit performance in speeding up the response, as reported in Figure 2A. In Figure 2C an example of the dynamics is reported, showing an acceleration of the response for both self-regulation strategies at an intermediate level of repression.

As the repression increases, the response acceleration to an activating signal relies more and more on an overshoot of protein concentration, well above the final steady state, both for iMSLs and tSLs. If the input signal has to drive the host gene to its functional steady state, a large overshoot can be unwanted since it represents an unnecessary metabolic cost and a possible source of toxic effects [57]. Thus, there is probably a limitation in the repression strength that can be applied to minimize the time separation between two functional steady states. On the other hand, a regime of strong repression makes the iMSL a pulse generator, a dynamical feature that can eventually lead to adaptation as will be discussed in a following section. The observation that the iMSL can function as a pulse generator is fully consistent with the fact that this circuit is a particular case of incoherent feedforwad loops that were indeed previously associated to pulse generation [30].

While the speeding up of activation is a property shared by incoherent feedforward loops (and specifically by iMSLs) and tSLs, an interesting peculiarity of the miRNA-mediated regulation in iMSLs emerges looking at the time required for *p* concentration to reach zero, starting from a constitutive level (Figure 2D reports an example of this dynamics). The iMSL can delay the switch-off kinetics of the host in the same repression regime where it can accelerate the activation and the extent of the introduced delay is again dependent on the mRNA to miRNA lifetime ratio (Figure 2B). This apparently counterintuitive behaviour can be easily qualitatively explained. When a constitutively expressed gene senses a transcription stop signal, the velocity of protein concentration decrease is established only by protein and mRNA degradation rates. For example, long living mRNAs are more persistent and can be translated for a longer time after the stop of transcription, and long living proteins are obviously more resilient. The same is true for tSLs or transcriptional feedforward loops: as the transcription is switched off, transcriptional repressors cannot exert any regulation and the protein level simply undergoes the exponential decrease dictated by mRNA and protein degradation. On the other hand, thanks to the post-transcriptional regulation in iMSLs, for each single miRNA that is degraded the still present mRNAs sense an increase in their translation rate. This increase clearly depends on the repression strength that miRNAs can exert (thus on the repression fold *p*_*ss*_/*p*_0_) and on the relative stability of mRNAs and miRNAs (*τ*_*r*_/*τ*_*s*_), as a fast miRNA turnover leads to a higher translation rate of the remaining mRNAs. Eventually, the general increase in mRNA translation rate for each miRNA degradation event can lead to a temporary boost in protein concentration above the original steady state (see Figure 2D).

It is important to notice that the dynamics just described can be altered if the miRNA acts mostly on mRNA degradation and depends on the timescale of miRNA-mRNA binding-unbinding. While the iMSL can always speed up the host gene expression in activation, the delay in the switch-off dynamics can vanish in case of fast miRNA-mediated induction of mRNA degradation. This issue is discussed in more details in the Additional file 1.

#### The circuit response dynamics can robustly keep the host gene in a high-expression state

**Figure 3:**
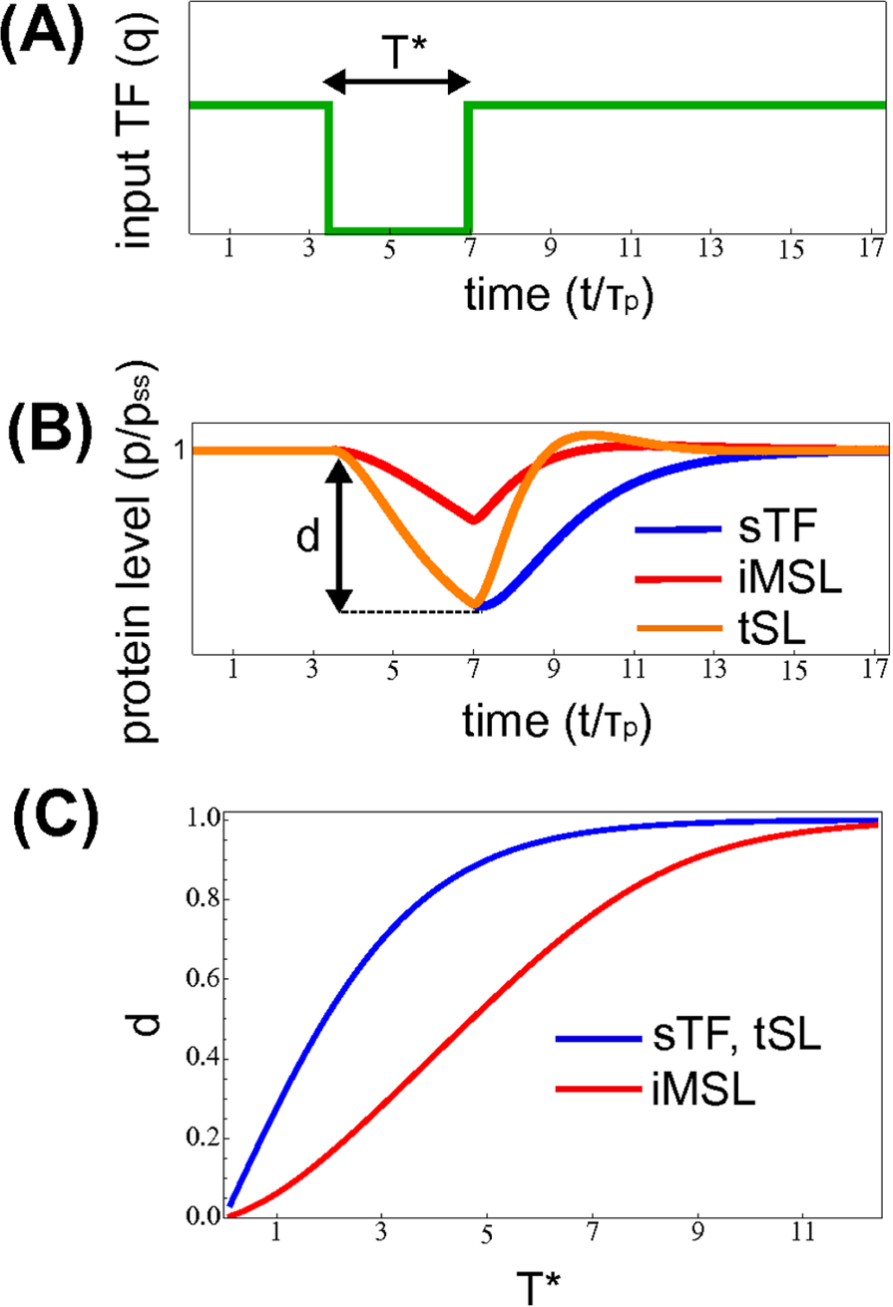
**miRNA-mediated self-loops can keep the host gene expression robustly in a ON-state.(A)** Schematic representation of a transient drop of the input TF *q* of duration *T*^∗^(time is in protein half-life units *τ*_*p*_) **(B)** Response of the three circuits to the temporary absence of signal depicted in A. The iMSL response (red line) is a slow protein concentration decrease, followed by a quick recovery of the ON-steady-state when the fluctuation is over. For a tSL (orange line) or a sTF (blue line) the switch-off dynamics is just due to mRNA and protein degradation. Even if the transcriptional negative feedback can accelerate the recovery, the distance *d* from the ON steady state that is reached by the target protein level during the temporary absence of the input activator is determined by the switch-off response. **(C)** The distance *d* from the ON steady-state reached by the target level is plotted as a function of the duration of the TF *q* absence (*d *= 1 when the protein level reaches zero). The iMSL circuit (red line) requires a more persistent absence of signal to show a significative reduction of the host protein product level with respect to the tSL or the sTF (blue line). The parameter values are the same of Figure 2, with comparable mRNA and miRNA stability (*τ*_*r*_/*τ*_*s *_= 1).

In the regime of comparable mRNA and miRNA lifetimes (red curves in Figure 2) the iMSL circuit can both accelerate the response to a switch-on signal and delay the switch-off kinetics. This alteration of the dynamics makes the host gene ON-state (expression at maximum rate) robust with respect to a transient fading of the input activating signal, as the one depicted in Figure 3A. In fact, the response to an input fluctuation toward zero is a slow protein concentration decrease, followed by a quick recovery of the ON-steady-state when the fluctuation is over (Figure 3B). Only a persistent absence of signal would cause a complete disappearance of the host protein product. In this way, the cell could prevent a drop in concentration of physiologically necessary proteins in merely presence of activator fluctuations. A resilient ON-state can be biologically important if it ensures the homeostatic protein level that must be robustly kept to mantain the correct phenotype or if the deactivation/reactivation is a costy process that have to be engaged only when undoubtedly necessary.

This property can be measured more quantitatively by the distance *d* from the ON-steady-state that is reached by the target protein level during a temporary absence of the input activator lasting a time *T*^∗^. As shown in Figure 3C, the iMSL regulation keeps the host gene protein product close to its steady state in presence of input fluctuations that would almost switch-off a gene transcriptionally self-regulated or constitutively expressed.

### Intronic microRNAs, targeting their host gene, can implement adaptation and Weber’s law

#### Adaptation

Adaptation is defined as the ability of a system to respond to a change in the input but subsequently return to the original level, even if the stimulus persists. Adaptation is ubiquitous in signaling systems. Examples of nearly perfect adaptation range from chemotaxis in bacteria [69] to sensor cells in higher organisms [70]. In all these systems, the benefit of adaptation can be summarized as the possibility of signal detection irrespective of the background level, thus widening the range of accessible signals and keeping the system robust in presence of perturbations.

Simple network topologies, as negative feedback loops with a buffering node or incoherent feedforward loops, can be at the basis of the cellular implementation of adaptation [71]. As a special case of incoherent feedforward loops, also iMSLs are expected to be suitable to implement adaptation. In this section, we investigate whether and in what conditions the minimal topology of a post-transcriptional self-regulation through intronic miRNAs can perform adaptation.

It is easy to show analytically (see Additional file 1) that in the regime of strong repression (*s*/*h*≫1 in the Michaelis-Menten function in Equation 2) the steady state of *p* concentration is independent of the input level *q*, which is clearly a hallmark of perfect adaptation [72]: after an eventual dynamical response to a change in *q*, the system always returns to its original equilibrium level. On the other hand, it is impossible to achieve such an independence on the input level at equilibrium using a tSL, as confirmed by the fact that in general circuits with just two molecular species are not adaptive [71].

**Figure 4:**
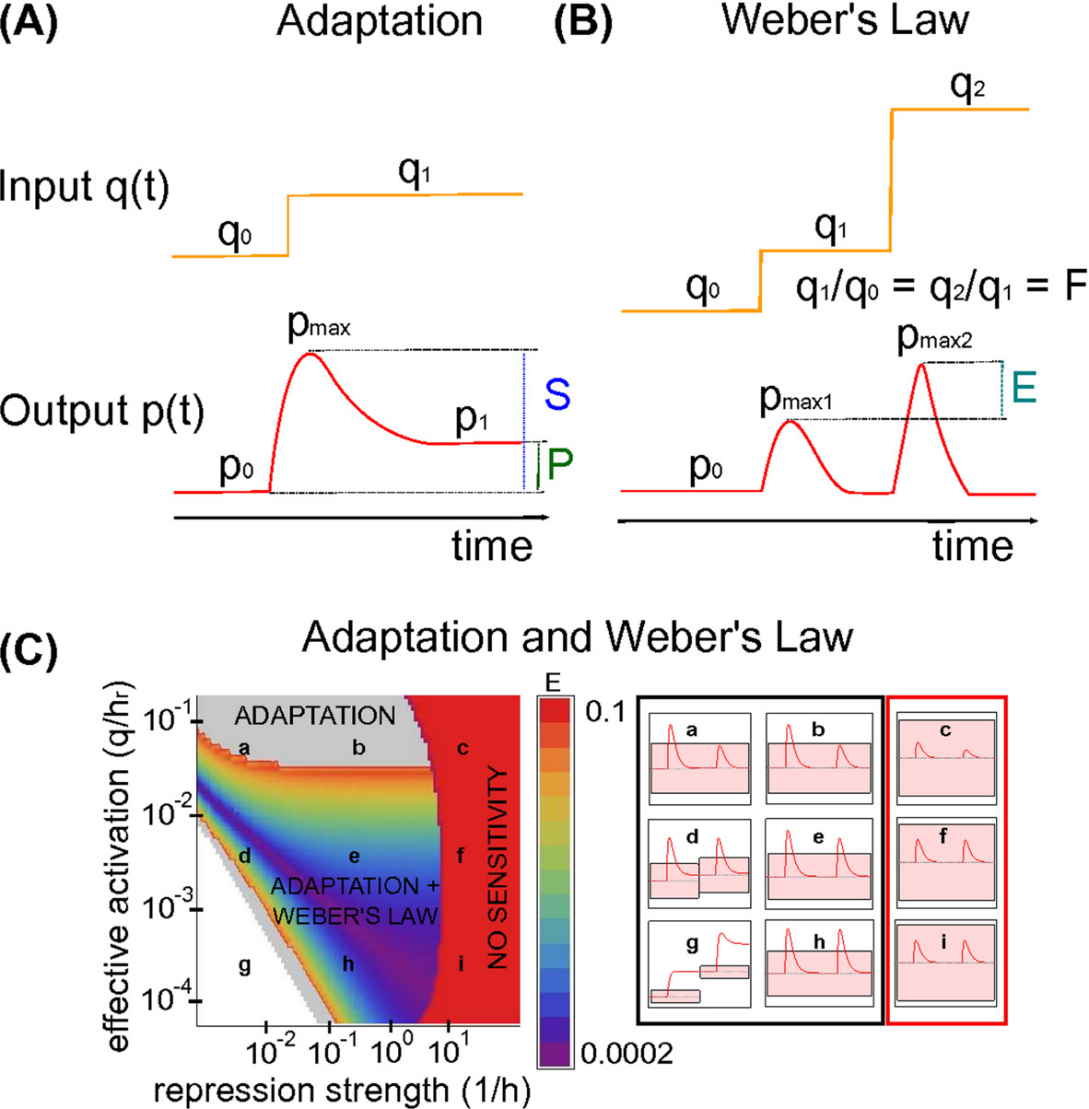
**Adaptation and Weber’s law implementation via intronic miRNA-mediated self-regulation.(A)** Schematic view of adaptative behaviour for a step-like input. *S* and *P* are the sensitivity and precision measures described in the main text. **(B)** Schematic view of Weber’s law implementation for a two-step input function. *E* is the error in fold-change detection, as defined in the main text. **(C)** A summarizing heat-map of the iMSL performances in implementing adaptation and Weber’s law, as a function of the effective activation *q*/*h*_*r *_and the repression strength 1/*h*. The grey region is the adaptive region (*P *> 10 and *S *> 2*CV*_*p*_), while the region where the system implements also Weber’s law (*E *< 0.1) is depicted with a color code representing the *E* value as reported in the legend. In the red zone the system is not sensitive enough to input variations (*S *< 2*CV*_*p*_). On the right, the target protein-level response to a two-step input is reported for the parameters values identified by the corresponding lower-case letters in the heat-map. The shaded regions correspond to the 2*CV*_*p *_sensitivity threshold, showing that for a too strong repression the circuit response cannot produce a signal beyond the noise level (plots in the red rectangle). The parameter setting is the following: mRNA and protein half-lifes as in Figure 2, miRNA half-life is *τ*_*s *_= 8 hours, *k*_*r *_= 2.12819*s*^−1^, *k*_*p *_= 0.048*s*^−1^, the input function starts from an initial value of *q*_0_ = 40 and makes two consecutive steps with fold-change *F *= 4.

More generally, we can evaluate the efficiency in performing adaptation giving the circuit a step function as input and calculating the two indexes of precision *P* and sensitivity *S*[71,73] represented in Figure 4A and defined by:

(4) $$\begin{array}{l} P\;=\;{\left|\frac{(p_{1}-p_{0})/p_{0}}{(q_{1}-q_{0})/q_{0}}\right|}^{-1}\\ S\;=\;\left|\frac{p_{\text{max}}-p_{0}}{p_{0}}\right|.\; \end{array}$$

*P* is a measure of the difference in the steady-state levels before and after the stimulus, therefore it is actually an estimate of the degree of adaptation. Following [71], we define the minimal threshold *P *> 10 to select adaptive circuits. A high value of *P* is not enough to define adaptation since it could merely be a consequence of complete insensitivity to input changes. Thus, it is necessary to check if the peak in *p*(*t*) concentration is an effective recognizable signal. This condition can be formalized requiring a sensitivity *S* above the noise level (*CV*_*p *_=* σ*_*p*_/〈*p*〉) of *p* at steady state, as can be calculated using the stochastic version of the model (see Additional file 1). In particular, we choose the threshold *S *> 2*CV*_*p*_ (assuming a noise in the input level *CV*_*q *_= 10*%*) to define a circuit “sensitive” to the input signal.

The general requirement of strong repression for an effective implementation of an adaptive response is in agreement with the results of an analogous theoretical analysis previously performed with detailed models of different feedforward loop circuits, including iMSLs [39]. In particular, the prediction that stronger repression should lead to better adaptivity was tested experimentally with a synthetic transcriptional feedforward loop, using the number of DNA molecules coding for the circuit to modulate the input signal [39].

#### Weber’s law

Certain adaptive systems, besides the ability to return to their original value after a signal response, present also a degree of response that is proportional to the relative change in the input signal and not to its absolute value. This feature is known as Weber’s law, originally introduced in the context of human sensory response. Recently, this dependence on input fold-change was demonstrated experimentally in eukaryotic signaling systems [74,75], and theoretically the feedforward loop topology was proposed as a candidate to Weber’s law implementation in gene regulatory networks [76]. Once again, it is natural to examine in what conditions also the minimal iMSL circuit can satisfy Weber’s law.

It is possible to show analytically (see Additional file 1) that iMSLs are responsive to input fold-change if three conditions are satisfied: 

• Strong repression: *s*/*h *≫ 1 ⇒* k*_*p*_(*s*) ≈* k*_*p*_*h*/*s*(condition for perfect adaptation),

• Almost linear promoter activation *k*_*r*_(*q*) ≈* qk*_*r*_/*h*_*r*_,

• Fast mRNA dynamics (short mRNA half-life with respect to miRNA and protein ones): *r*(*t*)→*r*_*ss*_.

As for the case of adaptation, we can quantify the efficiency in Weber’s law implementation for a generic set of biochemical parameters. To this aim, a two step input function is provided such that each step has the same fold-change but different background levels (see Figure 4B). As previously proposed [76], the error *E* in recognition of fold changes can be quantified using the difference in the response peaks:

(5) $$E=\left|\frac{p_{\text{max}2}-p_{\text{max}1}}{p_{\text{max}1}}\right|.$$

#### Parameter space of adaptation and Weber’s law

Using the observables defined in Equations 4 and 5, it is possible to explore the conditions in which adaptation and Weber’s law are successfully performed by iMSLs. An illustrative example is depicted in Figure 4C, where two effective parameters are varied: the effective promoter activation *q*/*h*_*r*_, and 1/*h* which measures the repression strength since *h* is the number of miRNAs necessary to reduce to one half the target translation rate. The grey region depicts the parameter space where precise adaptation is performed (*P *> 10), while in the excluded red region the dynamical response of the circuit is not able to go beyond the noise level (*S *< 2*CV*_*p*_). The *E* value is reported with the color code in the legend when the minimal condition *E *< 0.1 is satisfied, i.e the two steps of the input produce the same response within 10%. Adaptation and Weber’s law can be encoded by iMSLs in a parameter region that span several orders of magnitude of the effective parameters. Therefore, the only constraint is that the effective parameters have to approach the appropriate limits, without the need of fine-tuning.

It should be noticed that the general condition of strong repression required for both functions is limited by the circuit sensitivity. This is partially due to the fact that a too strong repression can rise the noise level of the circuit (see next section) making the achievement of a signal significantly above fluctuations harder.

It is interesting to consider what are the functional advantages that these two functions can provide to the host gene. Both adaptation and Weber’s law can bestow robustness to the expression program of the host gene. An expression state that is not influenced by constant inputs thanks to adaptation is robust with respect to the ubiquitous cell-to-cell variability in TF concentrations, but it is still responsive to signals that induce dynamical variations of TF levels. When additionally Weber’s law is implemented, also the dynamical response can be kept homogeneous in a cell population. In fact, in this case the response profile is only due to the input fold-change and not on its absolute value that is affected by the potentially variable background level [76]. Moreover, Weber’s law naturally encodes a noise filter. In fact, since the noise level is expected to scale with the background TF concentration, a dependence on fold-change can rescale appropriately the threshold at which the response is triggered, thus allowing a better signal/noise discrimination in different background conditions [76].

### Autoregulation via intronic microRNAs reduces the host gene expression fluctuations

**Figure 5:**
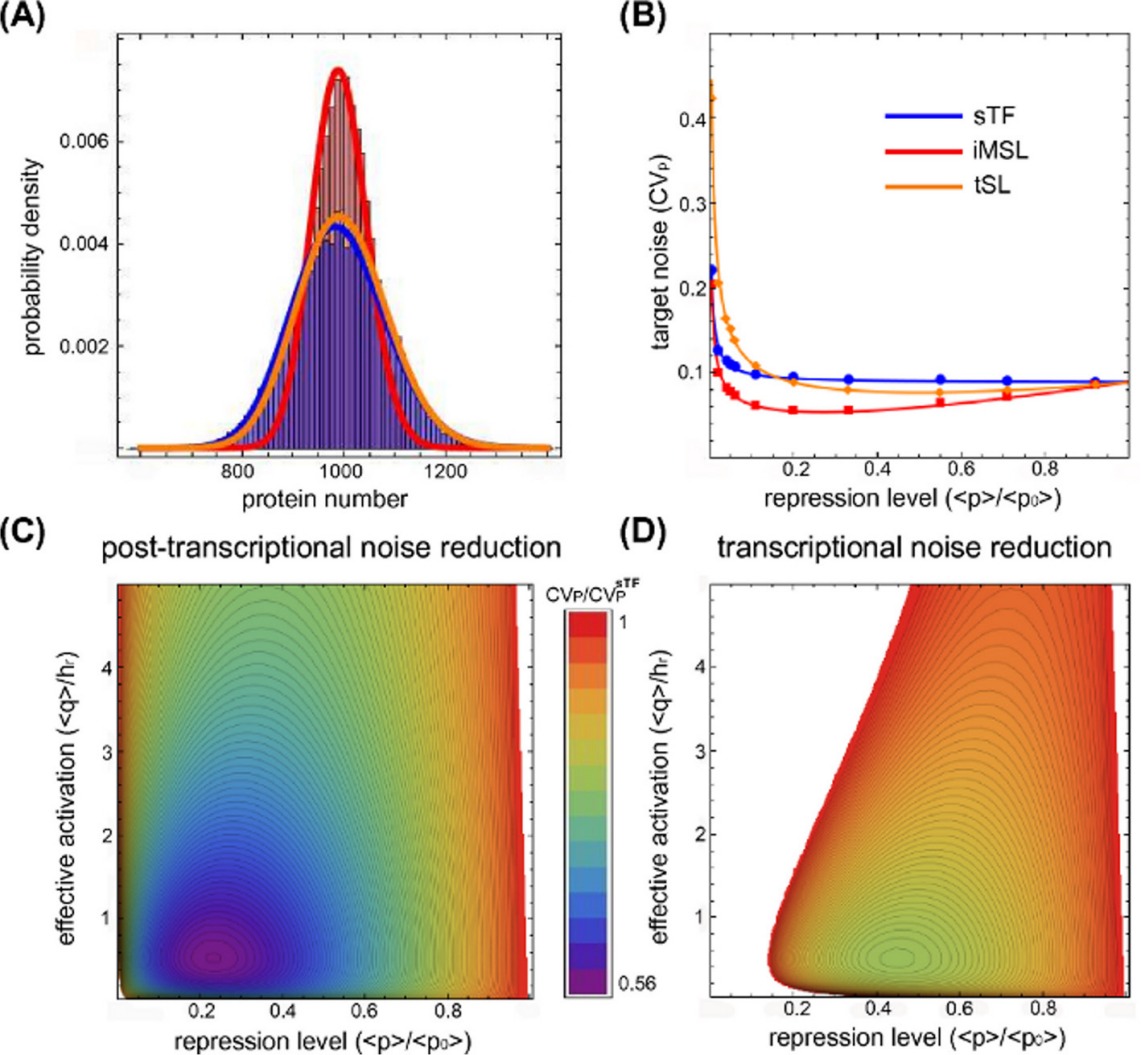
**Intronic miRNAs can buffer noise in host gene expression.(A)** An example of the target protein distributions for the three circuits (repression level 〈*p*〉/〈*p*_0_〉=0.2). Lines are gamma distributions with first two moments calculated analytically, while histograms are the result of Gillespie simulations. The distribution for the iMSL circuit (red line and histogram) is the narrowest, showing that, even if also a tSL (orange line and histogram) can reduce noise with respect to a sTF (blue line and histogram), the iMSL is outperforming. **(B)** Target noise *CV*_*p *_as a function of the repression level 〈*p*〉/〈*p*_0_〉 for the three circuits. Lines are analytical predictions, while dots are the result of Gillespie simulations. Given a noise level *CV*_*q *_≃ 0.2 in the upstream transcription factor, both the iMSL (red lines and dots) and the tSL (orange line and dots) shows a minimum of noise reduction with respect to the sTF (blue line and dots), but the level of fluctuations in the iMSL case is clearly lower. **(C,D)** Noise reduction on the target protein level achieved by the iMSL and the tSL respectively. The noise reduction $CV_{p}/CV_{p}^{\text{sTF}}$ (where $CV_{p}^{\text{sTF}}$ measures the fluctuations around the same mean level for a sTF) is evaluated at different degrees of transcriptional activation 〈*q*〉/*h*_*r *_and repression 〈*p*〉/〈*p*_0_〉. The same color gradient is used in both heat maps, showing that the iMSL reduces fluctuations on a larger parameter region and to a greater extent. In the white regions $CV_{p}>CV_{p}^{\text{sTF}}$. The parameter values are the following: mRNA half-life *τ*_*r *_=* τ*_*w *_= 30 minutes, protein half-life *τ*_*p *_=* τ*_*q *_= 1.5 hours, *k*_*w *_= 3.410^−3^*s*^−1^, *k*_*q *_= 8.710^−3^*s*^−1^, *k*_*r *_= 0.155*s*^−1^, *k*_*p *_= 4.810^−3^*s*^−1^(see Additional file 1 for the definition of kw and kq).

All the functions of iMSLs discussed so far contribute to enhance the robustness of the host gene expression. It is therefore natural to analyze a stochastic model of iMSLs to test directly their ability to filter out fluctuations. The stochastic analysis of the system is reported in detail in Additional file 1. The results in terms of noise-buffering properties at the steady state for the iMSL are similar to those obtained for the incoherent miRNA-mediated feedforward loops (see [44]). By filtering fluctuations propagating from the upstream TF, the steady-state target protein level achieved with an iMSL is less noisy than the same target amount obtained with a simple sTF or a tSL (Figure 5A,B). In particular, the target noise *CV*_*p*_ for the iMSL shows a U-shaped profile with a well defined minimum, thus allowing us to identify the parameter values that optimize the noise reduction properties (Figure 5B). This prediction could in principle be tested tuning the repression strength, as shown in [77] for a tSL. Also a tSL can in fact optimally filter noise for well defined values of repression strength [77-79], as shown in Figure 5B (orange dots and line). For this circuit the mechanism is well understood: an excessive increase of the repression strength (while potentially improving the noise reduction of the circuit) reduces the copy number of mRNAs and proteins with a consequent rise in intrinsic fluctuations (which can overcome attenuation). Thus, there is just a well defined range of repression strength for which the noise reduction is optimal, as shown in experiments [77].

It is interesting to notice that, even if iMSLs and tSLs show similar noise reduction properties, the miRNA-mediated self-regulation actually performs better than the transcriptional self-regulation. As it is possible to see in Figure 5A (where histograms and continuous lines are respectively the result of Gillespie simulations with full nonlinear dynamics and gamma distributions with analytically calculated moments), the probability distributions of the target protein level for the three circuits are different. Both autoregulatory circuits lead to a target distribution less sparse than a sTF, showing that they effectively reduce fluctuations, but the iMSL distribution is clearly more peaked than the tSL one. Similarly, both self-regulation strategies show an optimal noise buffering for an intermediate repression strength, but again the attenuation is larger in the miRNA-mediated case (see Figure 5B). This is more clearly shown in Figure 5C,D, where the noise reduction $CV_{p}/CV_{p}^{\text{sTF}}$ (with $V_{p}^{\text{sTF}}$representing the target noise in the case of a simple transcription unit producing the same mean amount of proteins) is reported for the two autoregulatory circuits. Noise reduction is explored for different levels of transcriptional activation (〈*q*〉/*h*_*r*_) and target repression (〈*p*〉/〈*p*_0_〉, where 〈*p*_0_〉 is the target mean value in absence of repression) to shed light into noise control and target suppression interdependence. Averages are here intended at steady state, thus the repression level measure 〈*p*〉/〈*p*_0_〉 for the stochastic model is perfectly equivalent to the one used in the response time analysis (see Figure 2). In the regime where the target is more sensitive to TF fluctuations, i.e. *q* is far from saturating the promoter, the iMSL can reduce the fluctuations up to a factor 0.5 (Figure 5C), while the tSL (Figure 5D) is much less effective. Moreover, the heat maps in Figures 5C,D indicate that the iMSL can buffer fluctuations over a wider range of conditions as well as to a greater extent.

As pointed out in [44], an optimal miRNA-mediated noise buffering does not necessarily require a strong repression. Indeed, Figure 5C shows that a reduction of the mean protein expression to 50% of its constitutive level is sufficient to reduce the noise by approximately 40%. This means that the intronic miRNA can keep the expression of its host gene in its homeostatic regime, while filtering out fluctuations, without exerting a strong reduction of its concentration. This result agrees well with the observation that miRNAs act often to fine-tune their targets rather than to switch them off completely [80].

### Sketch of the one-to-many topology-function map

This section summarizes the functions found to be associated to intronic miRNA-mediated self-loops into a qualitative “map of functions”, showing the different, although overlapping, ranges of biochemical parameters in which each specific function is optimized. The emerging map between parameter values and functions can be useful to understand the presence of the iMSL architecture in different biological contexts and gives general guidelines for the design of synthetic circuits with a desired behaviour, well beyond the simple suggestion of a network topology.

**Figure 6:**
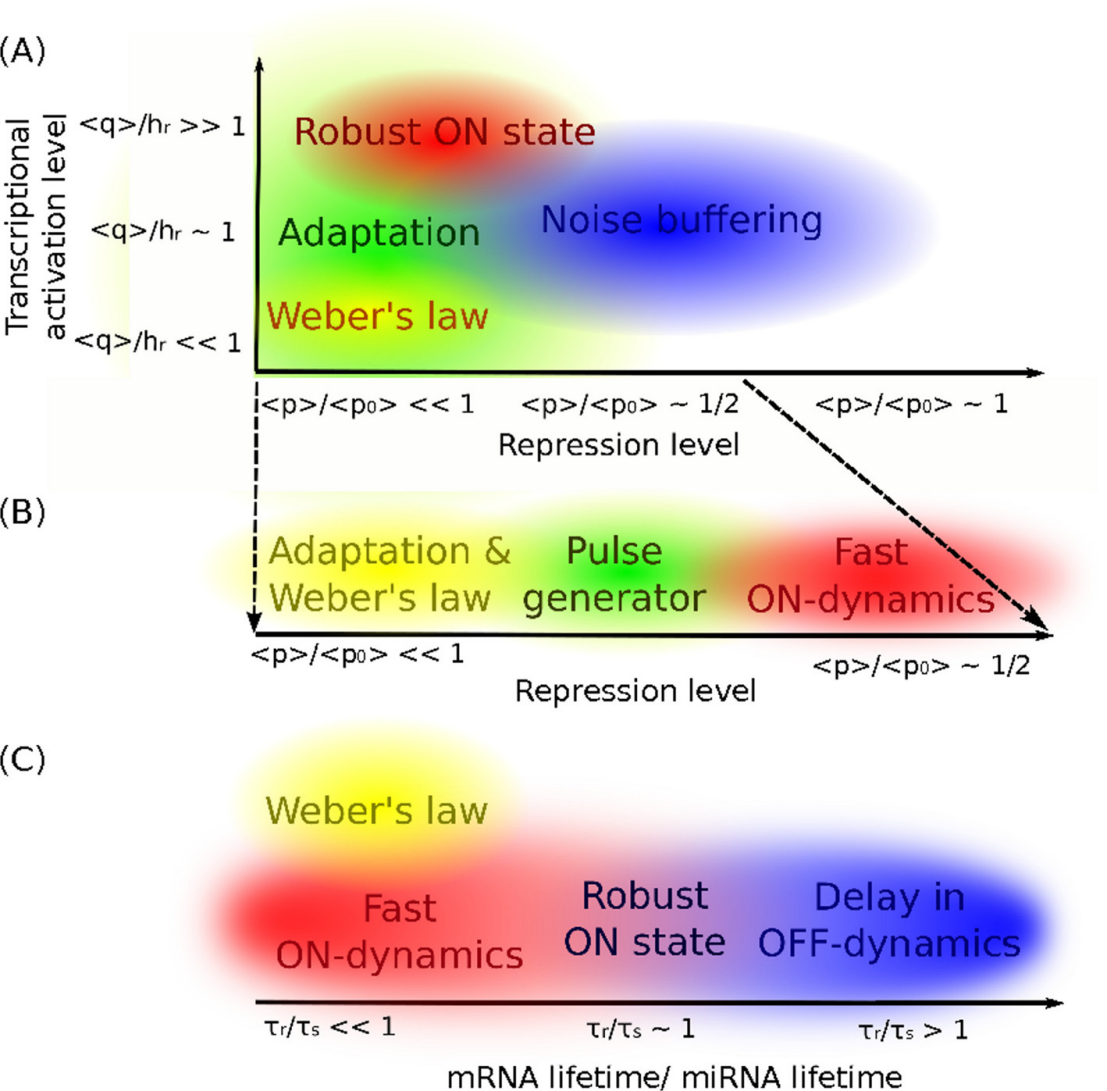
**Map of functions for an intronic miRNA-mediated self-loop.(A)** An ON-state of host gene expression is defined by full promoter induction (〈*q*〉/*h*_*r *_≫ 1), and a sufficiently strong miRNA repression (〈*p*〉/〈*p*_0_〉 < 0.5) can keep it robust in presence of temporary drops in the activator concentration. In the strong repression regime (〈*p*〉/〈*p*_0_〉 ≪ 1) adaptation can be observed, and for almost linear transcriptional activation (〈*q*〉/*h*_*r *_≪ 1) the host gene response can show an adaptive dynamics following Weber’s law. Fluctuations can propagate from the upstream TF more efficiently if the target promoter is highly sensitive to changes in TF level (〈*q*〉/*h*_*r *_≈ 1), thus in this parameter region noise buffering is more relevant, with a maximum in efficiency for intermediate repression (〈*p*〉/〈*p*_0_〉 ≈ 0.3). **(B)** A zoom on the strong repression region shows a transition between different dynamics. A step input can induce a fast transition of the host gene expression between two distinct steady states, but increasing further the repression the two steady states become progressively closer, up to their overlap when adaptation and Weber’s law are implemented. **(C)** The dynamics is strongly influenced by the relative stability of miRNAs and mRNAs. A short mRNA lifetime is a condition for Weber’s law implementation and contributes to the fast switch-on of the host gene expression. On the other hand, the delay in the switch-off dynamics is larger for short-living miRNAs. In the intermediate region, where the two half-lives are comparable, the trade-off between the two dynamical properties makes the highly-expressed state of the host gene robust with respect to fluctuations in the activator.

As Figure 6A shows, strong repression (〈*p*〉/〈*p*_0_〉≪1) is a general requirement for the implementation of adaptation and Weber’s law, the latter additionally requiring an almost linear activation of transcription (〈*q*〉/*h*_*r *_≪ 1). A sufficiently strong repression is also required to confer robustness to the high-expression state (induced by strong activation 〈*q*〉/*h*_*r *_≫ 1) of the host gene in presence of input temporary drops. On the other hand, for intermediate host activation, where the host gene promoter is highly sensitive to changes in the TF concentration, the iMSL can efficiently buffer fluctuations at steady state without the need of strong repression.

Looking at a finer scale the strong repression regime (〈*p*〉/〈*p*_0_〉 < 1/2), a smooth transition in the dynamical behaviour of the circuit can be observed (see Figure 6B). At first, the host gene is able to fastly transit between two well distinct steady states after induction. When the repression is further increased, this fast ON-activation relies increasingly on an overshoot well above the final equilibrium at which the dynamics asympotically relaxes. Therefore, the concentration profile resembles a pulse. Finally, for high enough repression the system returns to the initial steady state after the pulse, a necessary condition for the implementation of adaptation and Weber’s law.

The relative half-life of the molecules involved, in particular of miRNAs and mRNAs, is another ingredient that can strongly influence the dynamical behaviour (see Figure 6C). For example, a miRNA half-life comparable to the mRNA one allows a trade-off between acceleration of the ON-dynamics and delay of the OFF-dynamics, making the state of high expression of the host gene robust to fluctuations. On the other hand, mRNA lifetime must be short with respect to the other molecules lifetimes for a dynamical response following Weber’s law.

The present analysis of the iMSL functions considers the circuit as isolated, while realistically a single microRNA can target hundreds of genes. As recently pointed out, the degree of repression of a target depends on the level of expression of all possible target genes [45,81], since their mRNAs can dilute the pool of available miRNAs. Therefore, the expression profile of alternative miRNA targets is a variable that can potentially alter the dynamics of iMSLs (as shown for incoherent feedforward loops [44]), and thus have to be carefully taken into account in experimental tests on endogenous iMSLs.

### Identification of intronic miRNA-mediated self-loops in human

**Figure 7:**
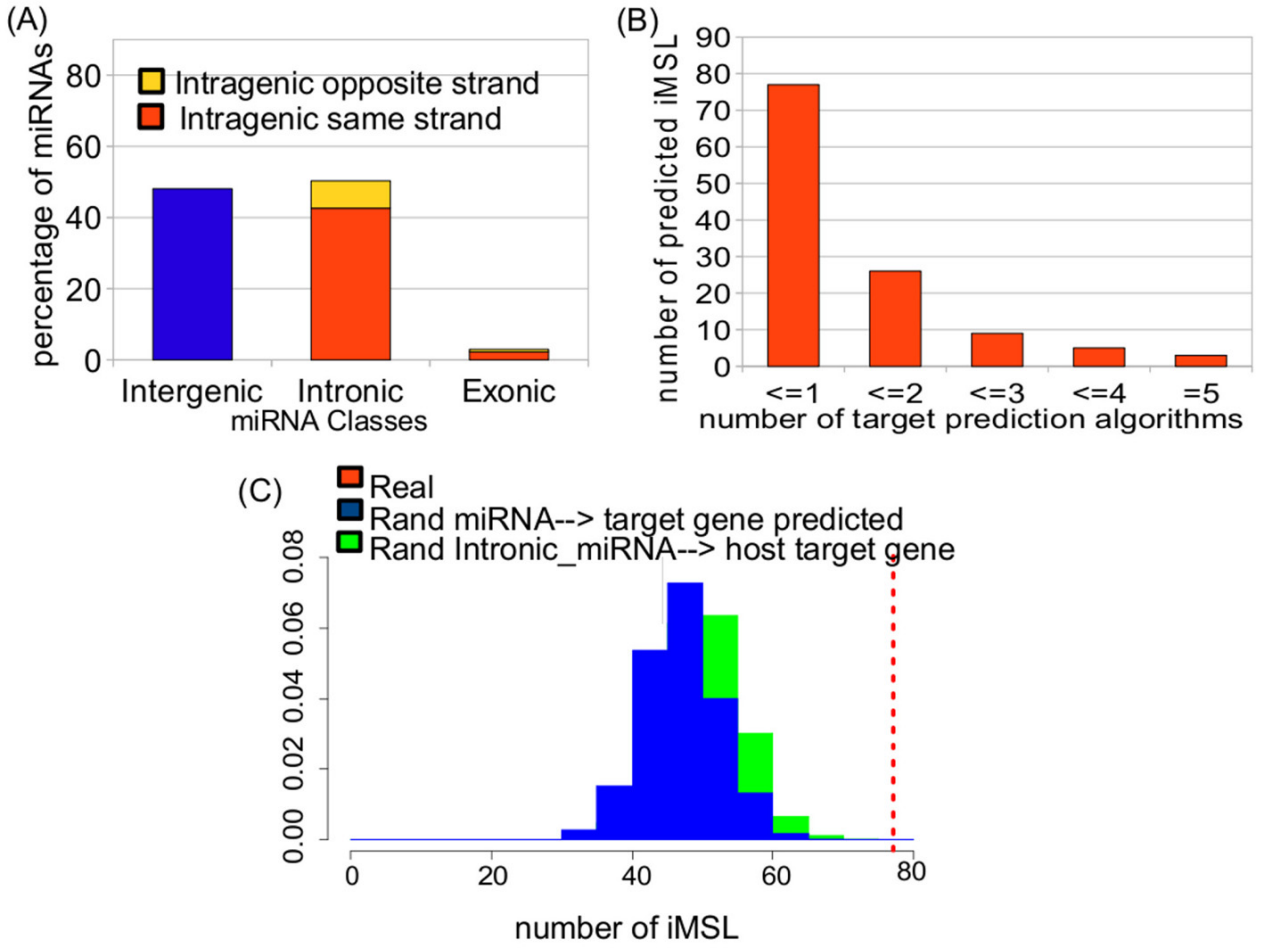
**Classification of miRNAs and randomization results.(A)** Classification of human miRNAs based on the percentage of intergenic and intragenic miRNAs. Intragenic miRNAs are divided in exonic and intronic miRNAs and each group can be further classified as same strand or opposite strand. All UTR miRNAs were included in the group of the exonic miRNAs. Data are reported in Table S1 of Additional file 1. **(B)** Number of intronic miRNA-mediated self-loops as a function of the number of target prediction methods in agreement: we found a total of 77 iMSLs predicted by at least one prediction methods, 25 of them are predicted by at least two different methods and only three of them are predicted by 5 different methods. **(C)** Results of the permutation test: the number of iMSLs in the human network is plotted as a dashed line alongside the distributions (normalized histograms) of the number of iMSLs found using the two randomization strategies (described in the main text) over 1000 experiment repetitions.

In this section, we briefly describe our bioinformatic search of iMSLs. Our main goal is to provide an updated list of candidates to eventually test our theoretical predictions. We performed a genome wide search of intronic miRNA-mediated self-loops along the lines of two papers which recently addressed the same issue [10,26]. The differences between our results and those quoted in [10,26] are mainly due to a different choice of the algorithms used to predict miRNA targets and in some cases to the use of updated versions of the corresponding databases. We identified the same strand intronic miRNAs using as reference the Ensembl (release 57) database. A summary of our results is reported in Table S1 of Additional file 1 and in Figure 7A, where the percentage of intergenic versus intragenic miRNAs is plotted and, for the intragenic ones, the relative ratio of exonic versus intronic miRNAs and of same-strand versus opposite strand is also reported. Target identification was performed using 8 different algorithms: TargetScan human v. 5.0 [3,82], miRanda - release 2008 [83,84], RNA22 [85], PITA-4way [86], MirTarget2 [87], PicTar [88], Diana microT v.3 [89] and TargetMiner v.1 [90]. In this respects, our analysis could be considered as a combination and extension of the one reported in reference [10] (where only the first 6 algorithms were used) and the study of [26] (where the authors used their own prediction algorithm). We were able in this way to find a total of 77 iMSLs confirmed by at least one algorithm (details are reported in Table S2 of Additional file 1). Since these algorithms are very different, we did not try to give an absolute score to our results, but ordered them starting from those which were assessed by the largest number of target prediction methods (Figure 7B and Table S2 of Additional file 1). Following a standard recipe (see [10] for a similar choice) we consider the number of different algorithms that agree on a certain target prediction as a measure of the confidence of such prediction. Interestingly, 28 of our iMSL agree with previous predictions of iMSLs [26] and for two of them an experimental validation of the miRNA-host gene regulation exist [26,28,29]. Moreover, a recent study [17] provides evidence supporting a feedback mechanism between miR-438 and IGF2, in agreement with our list of iMSLs predicted by only one method. In order to test if these iMSLs are over-represented we performed two independent enrichment tests. First we performed a reshuffling of the host genes while keeping miRNA target predictions unchanged. Second, we randomized the union of the datasets of miRNA target predictions obtained with the eight algorithms discussed above, keeping the host genes unchanged. In both cases we evaluated the *Z* score which turned out to be *Z *= 4.63 for the first test and *Z *= 5.52 for the second one (see the Methods section for more details). The results of the tests are plotted in Figure 7C. They suggest, in agreement with what already observed in [20,26], that this particular class of network motifs is under positive selection.

## Conclusions

This study presents a fairly comprehensive survey of the possibile functions associated to a miRNA-mediated circuit composed of one protein-coding gene (host gene) negatively regulated by a miRNA located in one of its introns. In particular, we have shown that, thanks to the miRNA-mediated self-regulation, the host gene expression responds to input changes with an altered timing, its response can be adaptative and follow Weber’s law, and fluctuations propagating from the upstream network are buffered at steady state. Each of these functions can confer robustness to the expression program of the host gene, suggesting that miRNA-mediated self-loops represents a simple homeostatic control. For example, adaptation makes the host gene expression level at equilibrium independent of the cell-to-cell variability of transcription factor expression without compromising its sensitivity to input changes. Similarly, the host expression dynamics, as modified by miRNA autorepression, can mantain the host gene in a high-expression state in the face of downwards fluctuations in activators’ concentration. The association of miRNA-mediated self-loops with functions with different specificities, but apparently the same final aim, suggests that, depending on the desired level of the host gene expression and on the type of fluctuations that have to be more frequently filtered out, the details of the regulatory interactions and the characteristics of the molecules involved could have been fine-tuned over evolutionary timescales accordingly. Such a fine-tuning of expression parameters has been shown to be possible even over short timescales in *in vitro* evolutionary experiments [91].

The comparison with an unregulated transcriptional unit and with a transcriptional negative feedback indicates that the specificities of miRNA regulation makes the post-transcriptional circuit better suited to implement a homeostatic control. This result is in line with the accumulating clues that miRNAs can help the cell to function reliably in presence of perturbations [31-34].

Finally, our systematic analysis of the constraints on biochemical parameters necessary to optimize each function can guide the realization of synthetic versions of miRNA-mediated self-loops, as well as contribute to the understanding of the role of their many occurrences in endogenous networks. In this perspective, we also provide a list of bioinformatically predicted miRNA-mediated self-loops in human for future experimental tests.

## Methods

### Stochastic simulations

Simulations were implemented by using Gillespie’s first reaction algorithm [92]. The reactions simulated are those presented in Figure 1 with additional transcription, translation and degradations for the input transcription factor *q*. Reactions that depend on a regulator were allowed to have as rates the corresponding full nonlinear functions. Results in Figure 5 are at steady state, which is assumed to be reached when the deterministic evolution of the system in analysis is at a distance from the steady state (its asymptotic value) smaller than its 0.05% (more than 10 times the protein half-life). Each data point or histogram is the result of 100000 trials.

### Bioinformatic methods

In order to identify human intragenic miRNAs, and associate them to their host genes, we used Ensembl-release 57 database. We collected the data for all human known protein coding genes (consisting in a total of 22.257 entries with a stable Ensembl Gene Identifier (ENSG)). For each gene we then retained for further analysis only the longest Transcript Identifier (ENST). The data on human miRNAs were extracted from Ensembl v.57, that includes miRBase v.13 (Table S1). To identify the iMSLs, we used eight tools for miRNA/target gene interaction predictions: TargetScan human v. 5.0 [3,82], miRanda - release 2008 [83,84], RNA22 [85], PITA-4way [86], MirTarget2 [87], PicTar [88], Diana microT v.3 [89] and TargetMiner v.1 [90]. To test the over-representation of the putative iMSLs, we performed two different types of randomization strategies. Specifically, we randomly permuted 1000 times the intronic miRNA/host-gene link and the union of miRNA/target gene interactions datasets predicted by the different algorithms. In both cases we created, according to the two reshuffling strategies, 1000 independent reshuffled copies of the original network. Then for each of them we evaluated the number of iMSLs confirmed by at least one algorithm and obtained in this way the two histograms plotted in Figure 7C. Then for both reshuffling strategies a *Z* score can be defined as : $Z=\frac{x-<x{>}_{r}}{\sigma_{r}}$, where *x* is the actual number of iMSLs in the network (i.e. 77), while <*x*>_*r*_ and *σ*_*r*_ are the mean value and the standard deviation of the distribution of iMSLs in the reshuffled samples. These *Z* scores turned out to be rather large: *Z *= 4.63 for the first test and *Z *= 5.52 for the second one (see Figure 7C). These values show that the number of iMSLs in the network is definitely larger than random and thus suggest, in agreement with what already observed in [20,26], that this particular class of network motifs is most probably under positive selection.

## Competing interests

The authors declare that they have no competing interests.

## Author’s contributions

CB, MO and MC designed research. CB, MO, ME, DC performed research. CB, MO, ME and MC wrote the paper. All authors read and approved the final manuscript.

## Supplementary Material

Additional file 1 **Supplementary information.** Single pdf file containing the details of our modeling strategy, a comparison with alternative models of microRNA regulation, and the results of our bioinformatic analysis. Click here for file
