# Supplementary material for: Gene autoregulation via intronic microRNAs and its functions
Source: BMC Syst Biol. 2012 Oct 10;6:131. doi: 10.1186/1752-0509-6-131 (PMC3534558; doi:10.1186/1752-0509-6-131)
Supplement: Additional file 1 — Supplementary information. Single pdf file containing the details of our modeling strategy, a comparison with alternative models of microRNA regulation, and the results of our bioinformatic analysis. [file 1752-0509-6-131-S1.pdf]

Gene autoregulation via intronic microRNAs and its functions - Supplementary Information

Carla Bosia*[1,2,†] , Matteo Osella*[3,4,†] , Mariama El Baroudi[5] , Davide Corá[2,6] , Michele Caselle[2,7]

[1] Human Genetics Foudation, V. Nizza 52a, I-10126, Torino, Italy. [2] Center for Complex Systems in Molecular Biology and Medicine, University of Torino, V. Accademia Albertina 13, I-10100 Torino, Italy. [3] Genomic Physics Group, UMR 7238 CNRS "Microorganism Genomics", 75006, Paris, France. [4] Université Pierre et Marie Curie, 15 rue de L'École de Médecine, 75006, Paris, France. [5] National Research Council (CNR), Institute of Informatics and Telematics (IIT) and Institute of Clinical Physiology (IFC), Laboratory for Integrative System Medicine (LISM), Via Giuseppe Moruzzi 1, I-56124, Pisa, Italy. [6] IRC@C: Institute for Cancer Research at Candiolo, School of Medicine, University of Torino, Str. Prov. 142, Km. 3.95, Candiolo I-10060, Torino, Italy. [7] Dipartimento di Fisica Teorica and INFN, University of Torino, V. Pietro Giuria 1, I-10125, Torino, Italy.

† Equal contributors

Email: Carla Bosia*- carla.bosia@hugef-torino.org; Matteo Osella*- matteo.osella@upmc.fr; Mariama El Baroudi - mariama.elbaroudi@iit.cnr.it; Davide Corá - davide.cora@ircc.it; Michele Caselle - caselle@to.infn.it;

*Corresponding author

Contents

1 Mean field analysis of the dynamics **3**

1.1 Simple transcriptional unit (sTF) 3

1.2 Intronic miRNA-mediated self loop (iMSL) 4

1.3 Transcriptional self regulation (tSL) 4

2 Conditions for adaptation and Weber's law implementation **5**

3 Noise buffering: master equation and generating function approach **7**

3.1 Intronic miRNA-mediated self loop (iMSL) 7

3.2 Simple transcriptional unit (sTF) 9

3.3 Transcriptional self-regulation (tSL) 10

4 Relation with other modeling strategies of miRNA-mediated regulation **10**

4.1 Molecular titration model 10

4.2 Relations between titration model and phenomenological models based on Michaelis-Menten functions 11
4.2.1 Adaptation and Weber's law conditions in the titration model 12
4.2.2 Comparison of the response times for different models of miRNA-mRNA interaction. . 13

5 Effects of the microRNA biogenesis process on the circuit functions. 15

6 Bioinformatic analysis - Supplementary tables 19

1 Mean field analysis of the dynamics

In this section the deterministic description of the regulatory circuits in analysis is reported in more details. On this description is based the evaluation of the response times presented in the main text.

1.1 Simple transcriptional unit (sTF)

We first consider the dynamics of a simple transcription unit (scheme in Figure 1B of the main text), for which the time evolution can be evaluated analytically in the two cases of interest: the dynamics of the switch-on and switch-off processes.

The system of equations representing the sTF dynamics is:

$$\begin{aligned} \frac{dr}{dt} &= k_r(q) - g_r r \\ \frac{dp}{dt} &= k_p r - g_p p, \end{aligned} \qquad (1)$$

where $k_r(q)$ is the nonlinear increasing function of the TF concentration q reported in the main text in equation 1.

With the target promoter exposed to full activation ($q/h_r \gg 1$), the transcription rate reduces to $k_r(q) \simeq k_r$ and it is possible to calculate how the final steady state is approached by the various molecular species starting from the initial condition $p(0) = r(0) = 0$:

$$\begin{aligned} \frac{r(t)}{r_{ss}} &= (1 - e^{-g_r t}) \\ \frac{p(t)}{p_{ss}} &= \frac{g_p(1 - e^{-g_r t}) - g_r(1 - e^{-g_p t})}{g_p - g_r}, \end{aligned} \qquad (2)$$

with

$$\frac{p(t)}{p_{ss}} = 1 - e^{-gt}(1 - gt) \;\; \text{if} \;\; g_r = g_p = g.$$

This expression can be simplified in the case of short-living mRNAs to $p(t)/p_{ss} \simeq (1 - e^{-g_p t})$ as reported in [1].

The response time T_{ON} is then defined by the equation $p(T_{ON})/p_{ss} = 0.5$. As can be seen in equations 2, T_{ON} does not depend on production rates (k_r and k_p) but only on the half-lives of mRNAs and proteins. Since T_{ON} for a sTF is independent of the final steady-state value of p, if molecule half-lives are kept constant, it can be used as null model for comparison with iMSLs and tSLs at different levels of repression, without the need of constraints on parameters.

Analogously, the response time T_{OFF} to a switch-off stimulus can be derived. In this case, the initial condition is the steady state given by a fully activated promoter $p(0) = p_{ss}$, and k_r is set to zero at $t = 0$. Again the dynamics depends only on the half-lives of mRNAs and proteins:

$$\begin{aligned} \frac{r(t)}{r_{ss}} &= e^{-g_r t}, \\ \frac{p(t)}{p_{ss}} &= \frac{g_p e^{-g_r t} - g_r e^{-g_p t}}{g_p - g_r}, \end{aligned} \tag{3}$$

where

$$\frac{p(t)}{p_{ss}} = e^{-gt}(1 + gt) \quad \text{if} \quad g_r = g_p = g.$$

The response time T_{OFF} is given by the condition $p(T_{OFF})/p_{ss} = 0.5$.

1.2 Intronic miRNA-mediated self loop (iMSL)

The deterministic description of the iMSL is given by equations 3 of the main text. With the condition $q/h_r \gg 1$, required to have the target promoter exposed to full activation, the transcription rate is at its maximum value k_r and the steady-state solution can be easily found:

$$\begin{aligned} s_{ss} &= \frac{k_r}{g_s} \\ r_{ss} &= \frac{k_r}{g_r} \\ p_{ss} &= \frac{r_{ss}}{g_p} \frac{k_p}{1 + s_{ss}/h}. \end{aligned} \tag{4}$$

On the other hand, the dynamics and thus the response times can be extracted with numerical integration. These response times have been normalized in the main text, in particular in Figure 2, with the response times of a sTF (calculated as explained in the previous section), so as to evaluate the differences in the dynamics with respect to a constitutive transcription unit.

1.3 Transcriptional self regulation (tSL)

The tSL dynamics is described by the two equations:

$$\frac{dr}{dt} = k_r(q,p) - g_r r$$
$$\frac{dp}{dt} = k_p r - g_p r, \tag{5}$$

where the transcription rate $k_r(q,p)$ is a product of two Michaelis-Menten-like functions: one corresponding to activation by the TF q and the other one taking into account the transcriptional self-repression. The choice of a simple product of functions implies the assumption of independent binding of the two regulators [2], which is probably the most common situation. Therefore, the form of the transcription rate is:

$$k_r(q,p) = k_r \left[\frac{q}{h_r + q} \frac{1}{1 + (\frac{p}{h_p})} \right]. \tag{6}$$

The condition $q/h_r \gg 1$ leads to the simplification $k_r(q,p) \simeq k_r(p) = k_r \frac{1}{1+(\frac{p}{h_p})}$. In this case the steady-state solution is:

$$r_{ss} = \frac{-g_p g_r h_p + \sqrt{g_p g_r h_p}\sqrt{g_p g_r h_p + 4k_p k_r}}{2 g_r k_p}$$
$$p_{ss} = \frac{\frac{-g_p h_p + \sqrt{g_p h_p}\sqrt{g_p g_r h_p + 4k_p k_r}}{\sqrt{g_r}}}{2 g_p}. \tag{7}$$

In order to compare the dynamics of tSLs with the iMSL one in un unbiased way, we impose a constraint on parameters in order to have the same steady state p_{ss} for the target protein level. This can be simply done by equating the values of p_{ss} in equations 7 and 4 so as to extract the constraint on the parameter h_p which sets the repression strength in the tSL depending on the repression strength h in the imLS:

$$h_p = \frac{g_s^2 h^2 k_p}{g_p g_r (g_s h + k_r)}. \tag{8}$$

Using this constraint, the response times for the tSL circuit can be evaluated numerically and directly compared with the response times of the iMSL circuit.

2 Conditions for adaptation and Weber's law implementation

As discussed in the main text, a necessary conditions to have perfect adaptation is the maintenance of a steady state independent of the input level, if this input is constant. In this way, the system can have a

dynamical response to input changes while returning to its initial condition once the input level is steady for a sufficiently long time.

In the case of iMSLs, a strong miRNA repression can make the circuit fulfill this condition. In the regime of strong repression $s/h \gg 1$, the translation rate simplifies to $k_p(s) \simeq hk_p/s$. The substitution of this expression in the equations describing the circuit dynamics (equations 3 of the main text), leads to a steady-state solution of the form:

$$\begin{aligned} s_{ss} &= \frac{k_r(q)}{g_s} \\ r_{ss} &= \frac{k_r(q)}{g_r} \\ p_{ss} &= \frac{k_p g_s}{h g_r g_p}. \end{aligned} \tag{9}$$

The steady state of the host-gene protein does not depend on the input level q, thus the iMSL circuit can in principle implement perfect adaptation.

Weber's law requires additionally that the peak of the dynamical response depends only on the fold-change of the input. Introducing the further assumption of an approximately linear transcriptional activation (i.e. the amount of TFs q is far from saturating the target promoter), the transcription rate becomes $k_r(q) \simeq \frac{k_r}{h_r} q$. Therefore, the two conditions of strong repression and approximately linear activation simplify the equations of the iMSL dynamics (equations 3 of the main text) to:

$$\begin{aligned} \frac{ds}{dt} &= \frac{k_r}{h_r} q - g_s s \\ \frac{dr}{dt} &= \frac{k_r}{h_r} q - g_r r \\ \frac{dp}{dt} &= h k_p \frac{r}{s} - g_p p. \end{aligned} \tag{10}$$

Assuming that the mRNA half-life is shorter than the other time scales in the system, a quasi-steady-state approximation can be used to further reduce the kinetic equations to:

$$\begin{aligned} \frac{ds}{dt} &= \frac{k_r}{h_r} q - g_s s \\ \frac{dp}{dt} &= \frac{k_p h k_r}{h_r g_r} \frac{q}{s} - g_p p. \end{aligned} \tag{11}$$

We can now define the following dimensionless variables:

$$
\begin{aligned}
t' &= g_s t && (12)\\
s' &= \frac{g_s h_r}{k_r q_0} s \\
p' &= \frac{g_r g_p}{g_s k_p h} \\
F &= q/q_0 \\
\phi &= g_s/g_p, && (13)
\end{aligned}
$$

where the input stimulus is represented by a change in the TF concentration from a basal level q_0 to a new level q (F is the fold-change). Equations 11 can be thus rewritten as :

$$
\begin{aligned}
\frac{ds'}{dt'} &= F - s' \\
\phi \frac{dp'}{dt'} &= \frac{F}{s'} - p'. && (14)
\end{aligned}
$$

This reformulation shows that the dynamics of the target protein depends only on the fold-change F in the input stimulus and not on its absolute value. Equations 14 are the analogous of the equations presented in [3] for the feed-forward loop circuit, adapted here to the iMSL case. Therefore, if the three conditions of strong repression, almost linear transcriptional activation and short mRNA half-life are satisfied, iMLs can implement Weber's law.

3 Noise buffering: master equation and generating function approach

3.1 Intronic miRNA-mediated self loop (iMSL)

This section briefly describes the procedure to calculate analitically the coefficient of variation CV_{x_i} for the molecular species x_i involved in the intronic miRNA-mediated self loop. The procedure can be similarly applied to the other two circuits (sTF and tSL).

For the stochastic analysis, the dynamics of transcription, translation and degradation of the TF is included explicitly. Therefore, the additional variable w, representing the TF mRNA number is added in the system description, as well as its transcription rate k_w, translation rate k_q and the degradation rates g_w and g_q. In this way, the noise level in the input signal can be naturally modulated changing the relative contribution of transcription and translation to the $\langle q \rangle$ steady-state level, as described in details in [4].

The following master equation describes the evolution of the probability to find in a cell exactly (w, q, s, r, p) molecules at a given time t:

$$
\begin{aligned}
\partial_t P_{w,q,s,r,p} = \quad & k_w(P_{w-1,q,s,r,p} - P_{w,q,s,r,p}) + k_q w(P_{w,q-1,s,r,p} - P_{w,q,s,r,p}) \\
& + k_r(q)(P_{w,q,s-1,r-1,p} - P_{w,q,s,r,p}) + k_p(s) r(P_{w,q,s,r,p-1} - P_{w,q,s,r,p}) \\
& + g_w \left[(w+1)P_{w+1,q,s,r,p} - wP_{w,q,s,r,p}\right] + g_q \left[(q+1)P_{w,q+1,s,r,p} - qP_{w,q,s,r,p}\right] \\
& + g_r \left[(r+1)P_{w,q,s,r+1,p} - rP_{w,q,s,r,p}\right] + g_s \left[(s+1)P_{w,q,s+1,r,p} - sP_{w,q,s,r,p}\right] \\
& + g_p \left[(p+1)P_{w,q,s,r,p+1} - pP_{w,q,s,r,p}\right], \qquad (15)
\end{aligned}
$$

where $k_r(q)$ and $k_p(s)$ have the functional form described in the main text.

In order to evaluate the noise level at steady state, given by the coefficient of variation $CV_{x_i} \equiv \sigma_{x_i}/\langle x_i \rangle$, for each molecular species x_i, it is necessary to find a closed expression for the first two moments of the above probability distribution at equilibrium. To this aim, it is sufficient to linearize the regulation functions $k_r(q)$ and $k_p(s)$ [4–6], and apply the moment generating function approach to the resulting master equation at equilibrium [7]. Even after the linearization, the system preserves a nonlinearity due to the term encoding the target translation, which still depends on both the number of mRNAs and miRNAs, but nonetheless the first two moments for the p distribution can be calculated. Using the linearization of the regulation functions:

$$
\begin{aligned}
k_r(q) &\simeq k_r(q)|_{\langle q \rangle} + \partial_q k_r(q)|_{\langle q \rangle}(q - \langle q \rangle) \\
k_p(s) &\simeq k_p(s)|_{\langle s \rangle} + \partial_s k_p(s)|_{\langle s \rangle}(s - \langle s \rangle), \qquad (16)
\end{aligned}
$$

the two rates can be redefined as:

$$
\begin{aligned}
k_r(q) &\simeq k_r^0 + q k_r^1 \\
k_p(s) &\simeq k_p^0 - s k_p^1, \qquad (17)
\end{aligned}
$$

with:

$$
\begin{aligned}
k_0^r &= k_r(q)|_{\langle q\rangle} - \partial_q k_r(q)|_{\langle q\rangle}\langle q\rangle \\
k_1^r &= \partial_q k_r(q)|_{\langle q\rangle} \\
k_0^p &= k_p(s)|_{\langle s\rangle} + \partial_s k_p(s)|_{\langle s\rangle}\langle s\rangle, \\
k_1^p &= -\partial_s k_p(s)|_{\langle s\rangle}, \qquad (18)
\end{aligned}
$$

By defining the generating function:

$$
F(z_1, z_2, z_3, z_4, z_5) = \sum_{w,q,s,r,p} z_1^w\ z_2^q\ z_3^s\ z_4^r\ z_5^p\ P_{w,q,s,r,p}, \qquad (19)
$$

and using the linearized regulation functions, equation 15 can be converted in the following second-order partial differential equation:

$$
\begin{aligned}
\partial_t F = k_w(z_1F - F) + k_q z_1(z_2\partial_{z_1}F - \partial_{z_1}F) + k_r^0(z_4F - F) \\
+k_r^1 z_2(z_4\partial_{z_2}F - \partial_{z_2}F) + k_p^0 z_4(z_5\partial_{z_4}F - \partial_{z_4}F) \\
-k_p^1 z_3 z_4(z_5\partial_{z_3,z_4}F - \partial_{z_3,z_4}F) + g_w(\partial_{z_1}F - z_1\partial_{z_1}F) \\
+g_q(\partial_{z_2}F - z_2\partial_{z_2}F) + g_s(\partial_{z_3}F - z_3\partial_{z_3}F) \\
+g_r(\partial_{z_4}F - z_4\partial_{z_4}F) + g_p(\partial_{z_5}F - z_5\partial_{z_5}F). \qquad (20)
\end{aligned}
$$

The differentiation of 20 at the steady state leads to equations for successively higher moments thanks to the following properties of the moment generating function: $F|_1 = 1$, $\partial_{z_i}F = \langle x_i\rangle$ and $\partial_{z_i}^2 F = \langle x_i^2\rangle - \langle x_i\rangle$ (where $|_1$ denotes the evaluation of F at $x_i = 1$ for all i). Differentiating up to the fourth moments, the analytical expression for $CV_{x_i} = \frac{\sigma_{x_i}}{<x_i>}$ can be obtained (see [4] for a more exhaustive and detailed analysis), and thus also the noise level of the host-gene protein product used in the main text.

3.2 Simple transcriptional unit (sTF)

The stochastic analysis of the sTF can be performed as explained in the previous section. We just report here the corresponding master equation:

$$
\begin{aligned}
\frac{\partial P_{w,q,r,p}}{\partial t} = k_w(P_{w-1,q,r,p} - P_{w,q,r,p}) + k_q w(P_{w,q-1,r,p} - P_{w,q,r,p}) \\
+k_r(q)(P_{w,q,r-1,p} - P_{w,q,r,p}) + k_p r(P_{w,q,r,p-1} - P_{w,q,r,p}) \\
+g_w[(w+1)P_{w+1,q,r,p} - wP_{w,q,r,p}] + g_q[(q+1)P_{w,q+1,r,p} - qP_{w,q,r,p}] \\
+g_r[(r+1)P_{w,q,r+1,p} - rP_{w,q,r,p}] + g_p[(p+1)P_{w,q,r,p+1} - pP_{w,q,r,p}]. \qquad (21)
\end{aligned}
$$

3.3 Transcriptional self-regulation (tSL)

The master equation for the transcriptional self-regulation (tSL) is:

$$\frac{\partial P_{w,q,r,p}}{\partial t} = k_w(P_{w-1,q,r,p} - P_{w,q,r,p}) + k_q w(P_{w,q-1,r,p} - P_{w,q,r,p})$$
$$+k_r(q,p)(P_{w,q,r-1,p} - P_{w,q,r,p}) + k_p r(P_{w,q,r,p-1} - P_{w,q,r,p})$$
$$+g_w[(w+1)P_{w+1,q,r,p} - wP_{w,q,r,p}] + g_q[(q+1)P_{w,q+1,r,p} - qP_{w,q,r,p}]$$
$$+g_r[(r+1)P_{w,q,r+1,p} - rP_{w,q,r,p}] + g_p[(p+1)P_{w,q,r,p+1} - pP_{w,q,r,p}]. \qquad (22)$$

In order to calculate CV_p with the moment generating function approach, it is necessary to define the linearization of the function $k_r(q,p)$ shown in equation 6. As described in [4], we can linearize it as:

$$k_r(q,p) \simeq k_r(q,p)|_{\langle q\rangle,\langle p\rangle} + \partial_q k_r(q,p)|_{\langle q\rangle,\langle p\rangle}(q - \langle q\rangle)$$
$$+ \partial_p k_r(q,p)|_{\langle q\rangle,\langle p\rangle}(p - \langle p\rangle), \qquad (23)$$

and thus obtain a transcription rate of the form $k_r(q,p) \simeq k_r^0 + k_r^1 q - k_r^2 p$. Using this linearization and the moment generating function approach, the analytical expressions of $\langle p\rangle$ and CV_p can be obtained as described in previous sections.

4 Relation with other modeling strategies of miRNA-mediated regulation

4.1 Molecular titration model

A mathematical model of miRNA-mRNA interaction was previously proposed to describe sRNA regulation in bacteria [8]. This model takes into account the physical coupling between miRNAs and mRNAs explicitly with a simple titration mechanism: a miRNA can form a complex with a target mRNA, degrade it and then eventually be available again to target other mRNAs. A parameter α is introduced to measure the probability of miRNA recycling after target degradation induced by mRNA-miRNA coupling. Thus, α represents the degree of "catalyticity" of miRNA regulation, with $\alpha = 0$ for perfect catalytic action, while $\alpha = 1$ for stoichiometric action.

Applying this modeling strategy to the iMSL circuit, the following system of differential equations is obtained:

$$
\begin{aligned}
\frac{ds}{dt} &= k_r(q) - g_s s - (k_+ rs - k_- c) + (1-\alpha)\beta c \\
\frac{dr}{dt} &= k_r(q) - g_r r - (k_+ rs - k_- c) \\
\frac{dc}{dt} &= k_+ rs - k_- c - \beta c \\
\frac{dp}{dt} &= k_p r - g_p p. \qquad (24)
\end{aligned}
$$

In this equations, c is the concentration of the miRNA-mRNA complex, k_+ is the probability of miRNA-mRNA association and k_- the probability of dissociation of the complex c, that can degrade with rate β. An analogous model of miRNA regulation have been used to describe the results of single-cell experiments in mammalian cells [9], with the additional assumption of slow miRNA turnover, thus neglecting the dynamics of miRNA transcription and degradation.

4.2 Relations between titration model and phenomenological models based on Michaelis-Menten functions

If the coupling of miRNAs and mRNAs is fast, or if the interest is on steady state properties, the c dynamics can be equilibrated in equations 24:

$$
\begin{aligned}
\frac{ds}{dt} &= k_r(q) - g_s s - \alpha k_{rs} rs \\
\frac{dr}{dt} &= k_r(q) - g_r r - k_{rs} rs \\
\frac{dp}{dt} &= k_p r - g_p p, \qquad (25)
\end{aligned}
$$

with $k_{rs} = \beta k_+/(k_- + \beta)$. miRNA regulation is often distinguished from sRNA one because of the efficient recycling (i.e. catalytic interaction). In particular, in the case of $\alpha \simeq 0$, the molecular concentrations at steady state are:

$$
\begin{aligned}
r_{ss} &= \frac{k_r}{g_r} \frac{1}{1 + k_{rs} s/g_r} \\
p_{ss} &= k_p \frac{k_r}{g_r} \frac{1}{1 + k_{rs}/g_r \; s} = r_0 k_p \frac{1}{1 + k_p s/h}, \qquad (26)
\end{aligned}
$$

where $r_0 = k_r(q)/g_r$ is the steady state for a constitutive mRNA, while $h = g_r/k_{rs}$. This expression for the target protein concentration shows the equivalency to the model of miRNA inhibition of target

translation used in the main text. In fact, the same steady state can be obtained using an effective Michaelis-Menten function of miRNA concentration as target translation rate (as in equations 3 of the main text), with an effective dissociation constant $h = g_r/k_{rs}$. Therefore, in the limit of high miRNA recycling the steady state properties of a titration model are completely equivalent to an effective model of nonlinear miRNA action on target translation rate.

The miRNA regulation can also be modeled using an effective nonlinear function in the mRNA degradation term, thus assuming that miRNA regulation acts mainly on the stability of mRNAs rather than on their translation efficiency. In this case, the equations describing the iMSL circuit dynamics are:

$$\begin{aligned}\frac{ds}{dt} &= k_r(q) - g_s s \\ \frac{dr}{dt} &= k_r(q) - (g_r + g_{max}\frac{s}{h+s})r \\ \frac{dp}{dt} &= k_p r - g_p p. \end{aligned} \tag{27}$$

The miRNA action is represented by adding to the basal rate of mRNA degradation g_r (in absence of miRNAs) an increasing function of miRNA concentration, where g_{max} is the maximum possible increase of the degradation rate (if $s \to \infty, g_r(s) \to g_r + g_{max}$) and h is the dissociation constant of miRNA-mRNA interaction. It's easy to see that in the case of strong enough repression, for which $s/h \gg 1$, the equation for r in 27 can be recasted as:

$$\frac{dr}{dt} = k_r(q) - g_r r - \frac{g_{max}}{h} rs, \tag{28}$$

making clear the relation between this description and the titration model with fast binding/unbinding of mRNA and miRNAs and high cataliticity, i.e. equations 25 with $\alpha \simeq 0$ and $k_{rs} = g_{max}/h$.

4.2.1 Adaptation and Weber's law conditions in the titration model

As shown in section 2, the iMSL can perform adaptation in the regime of strong miRNA-mediated repression. In the context of the titration model with high catalyticity ($\alpha \simeq 0$), strong repression implies that the degradation of mRNAs is dominated by miRNA regulation. Therefore, we can approximate equations 25 with:

$$\begin{aligned}\frac{ds}{dt} &= k_r(q) - g_s s \\ \frac{dr}{dt} &\simeq k_r(q) - k_{rs} r s \\ \frac{dp}{dt} &= k_p r - g_p p. \end{aligned} \tag{29}$$

The steady state solution for the target protein is:

$$p_{ss} = \frac{k_p g_s}{k_{rs} g_p}. \tag{30}$$

This expression is independent on the input level q, showing that the condition for adaptation is satisfied in the strong repression regime also for this alternative modeling strategy of miRNA regulation, if the miRNA recycling is sufficiently efficient as it is expected for miRNA regulation in higher eukaryotes. Given the steady-state equivalency (shown in the previous section) between the titration model with $\alpha \simeq 0$ and a phenomenological model of mRNA degradation induction, the addition of iMSLs to the list of adaptive circuits seems robust and model-independent.

Starting from equations 29, in which there is also the implicit assumption of fast mRNA-miRNA binding/unbinding, also the conditions for Weber's law implementation can be examined in the context of the titration model. As previously discussed, the additional requirements with respect to adaptation are an almost linear transcriptional activation and a fast mRNA dynamics. With these two constraints the dynamic equations become:

$$\begin{aligned}\frac{ds}{dt} &= \frac{k_r}{h_r} q - g_s s \\ \frac{dp}{dt} &= \frac{k_p k_r}{h_r k_{rs}} \frac{q}{s} - g_p p. \end{aligned} \tag{31}$$

These two equations have exactly the same form of equations 11, thus can be similarly reformulated in terms of adimensional variables, showing that the p dynamics depends only on the input fold-change.

4.2.2 Comparison of the response times for different models of miRNA-mRNA interaction.

A direct comparison of the response times for the different models of miRNA-mRNA interaction is made difficult by the higher number of parameters that are present in the titration model (equations 24) with respect to the phenomenological model presented in the main text. The way in which the two models can be

constrained to have the same level of target protein at equilibrium, in order to make an unbiased comparison, is indeed quite arbitrary. In particular, the timescale of the binding/unbinding of mRNAs and miRNAs in the c complex can strongly influence the dynamics.

For example, for fast binding/unbinding (as in equation 25 and equivalently in equations 27), the iMSL reduces the time required to switch-on the host-gene expression, but also accelerates the switch-off dynamics. The reduced effective mRNA half-life drives a fast drop in mRNA concentration, and thus of proteins. Therefore, in this conditions the iMSL is not effective in keeping the ON-state robust with respect to fluctuations in the activator level. However, the opposite case of a long-living mRNA-miRNA complex have dynamical properties more similar to those of the phenomenological model presented in the main text. The simplified situation of mRNA sequestration in long-living miRNA-mRNA complexes from which mRNAs cannot be translated, but mRNAs and miRNAs degrade with their natural rates, can be considered for a comparison. The iMSL dynamics in this case is described by the equations:

$$\begin{aligned}
\frac{ds}{dt} &= k_r(q) - g_s s - (k_+ rs - g_r c) \\
\frac{dr}{dt} &= k_r(q) - g_r r - (k_+ rs - g_s c) \\
\frac{dc}{dt} &= k_+ rs - (g_r + g_s)c \\
\frac{dp}{dt} &= k_p r - g_p p.
\end{aligned} \tag{32}$$

This model can be easily constrained to have the same p_{ss} solution of the model presented in the main text, acting on the parameter k_+. The results of the analysis of the response times are qualitatively equivalent to those presented in the main text, although a miRNA-mediated repression of translation seems quantitatively more efficient in locking the ON-state of the host-gene expression.

Therefore, while the acceleration of the host-gene activation is a result independent of the type of miRNA repression, the delayed switch-off kinetics is expected to be observed for miRNAs repressing target translation or miRNAs that can bind mRNAs in sufficienlty stable complexes. A stoichiometric repression based on coupled miRNA-mRNA degradation, like the one reported for sRNAs in bacteris [8], or a nonlinear induction of mRNA degradation could instead change the switch-off dynamics. A better experimental understanding of the mechanisms of miRNA-mRNA interaction in the specific case in analysis and measurments of the model parameters are thus required to fully address the details of the dynamics of miRNA-mediated circuits with a quantitative mathematical description.

5 Effects of the microRNA biogenesis process on the circuit functions.

As discussed in the main text, the multi-step process of miRNA biogenesis can influence the dynamics of the iMSL circuit. In particular, the time needed for the miRNA to be processed, exported, loaded in the RISC complex, and in general to become active may introduce a delay between its transcription and its effect on targets. Therefore, even if the mRNA and the miRNA come from the same transcript, a certain amount of time can be required before observing miRNA repression, thus altering the correlation in mRNA and its intronic miRNA dynamics that in principle follows from their co-transcription. There is evidence that intronic miRNAs can be cleaved by the Drosha enzyme before splicing [10], thus limiting the delay introduced by the nuclear processing. However, even in this case the subsequent steps of cytoplasmic processing and incorporation into RISC can potentially delay miRNA activation. While in the model presented in the main text miRNAs are supposed to act on their target instantaneously, in this section we present the results of a numerical analysis performed taking into account the time-lag that can arise from miRNA processing in a phenomenological way. In numerical simulations, the time-delay has been inserted in the Michaelis-Menten function of regulation of the target translation, mimicking the time required for miRNA activation. To the best of our knowledge there are no systematic measurements of the timescales of the several miRNA maturation processes, therefore we consider different possible delay values.

The response times to an activating/deactivating input signal are significantly dependent on the delay (see Figure 1A and B). In case of activation, the longer is the time required to have active miRNAs, the more the protein concentration can rise in absence of any repression. Therefore, the response time T_{ON} is expected to decrease with the delay as indeed shown in Figure 1A. When the time gap between miRNA transcription and activation is large enough to allow the target protein concentration to reach the equilibrium in absence of repression, the response time is actually the one of a constitutive gene approaching its steady state. This explains why curves corresponding to different large delay values tend to overlap in Figure 1A.

As discussed in the main text, in presence of a deactivating signal the switch off dynamics of iMLS is slower than the deactivation dynamics of a constitutive gene. This is due to the fact that after the transcription stop, for each single miRNA that is degraded, the still present mRNAs sense an increase in their translation rate, thus slowering the dynamics with respect to an exponential decrease due to protein and mRNA degradation. In presence of a time-consuming miRNA processing, while degradation events of active miRNAs are still expected to increase the effective target translation rate, at the same time miRNAs that complete the processing steps can increase the pool of active miRNAs. Therefore, the slowdown of the deactivation dynamics is expected to be weaker in presence of a long biogenesis process, as indeed captured by our

phenomenological model (see Figure 1B).

For the sake of completeness, we also report the analysis of the case in which miRNA maturation is faster than mRNA processing. This situation can emerge for example in presence of a slow mRNA export with respect to the miRNA one, thus leading to a faster increase of miRNA concentration in the cytoplasm after transcription activation with respect to the mRNA concentration. The response times correponding to this case (blue dashed lines in Figure 1A and B) show that, as intuitively expected, an eventual delay in the mRNA dynamics (inserted in the model as a time-lag between transcription and translation) has opposite effects on the circuit response with respect to a delay due to miRNA maturation.

The alteration of the dynamics that can arise from miRNA biogenesis has also consequences on the adaptation response of the iMSL circuit. The adaptation precision P (defined in the main text) is not affected by the presence of the delay, since it is a property that follows from the steady-state protein dependence on the input level. On the other hand, as previously discussed for the reponse times, the presence of a delay in miRNA activation allows the protein concentration to reach higher levels in presence of a step activating signal. Therefore, the peak of the dynamical response to a step input, before relaxation to the final steady state, can be significantly increased by the delay, as shown in Figure 1C. In other words, the delay can increase the sensitivity S (see definition in the main text) without affecting the adaptation precision. However, the increased sensitivity must be compared to the noise level ($CV_p = \sigma_p / \langle p \rangle$) of p at steady state, to measure how much the produced signal is above the steady-state fluctuations. Noise is expected to increase in presence of a delay in miRNA activation. In fact, the noise buffering property of the iMSL relies on the correlation between miRNA and mRNA fluctuations, and this correlation can be disrupted by the presence of a delay, as we have previously shown for miRNA-mediated feedforward loops [4]. We performed stochastic simulations in order to compare sensitivity and fluctuations for different delay values. The results of this comparison are shown in Figure 1D, where the sensitivity normalized with $2CV_p$ is depicted as a function of the delay time. With this normalization the response signal can be considered above the noise level (following the definition in the main text) if the normalized sensitivity is greater than 1. The reduced efficiency in noise buffering does not compensate the increased peak amplitude of the protein concentration response, thus making the normalized sensitivity an increasing function of the delay time. This result implies that a difference in the timescales of miRNA and mRNA dynamics due to a delay in miRNA activation has a constructive effect on the implementation of adaptation by iMSLs.

The processes of miRNA and mRNA biogenesis can also induce different effective synthesis rates of mature mRNAs and active miRNAs, for example if only a certain percentage of transcribed miRNAs can complete

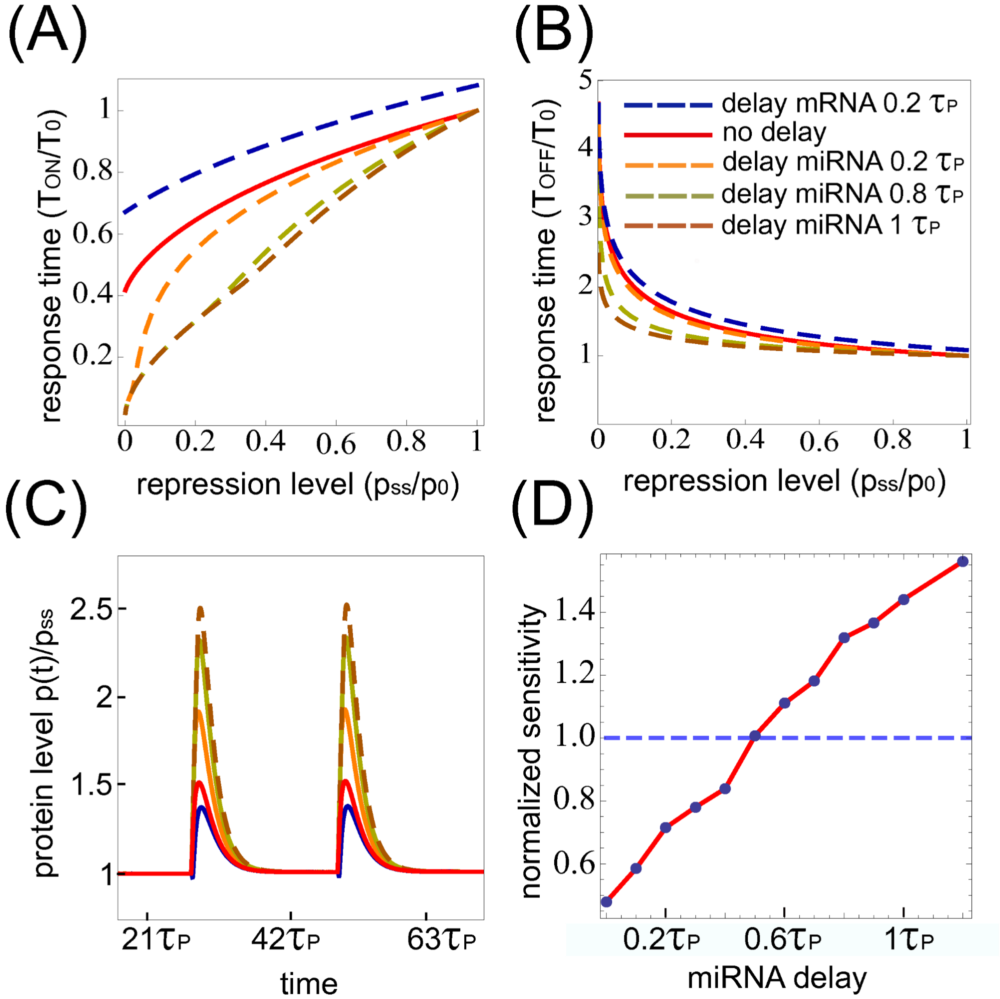

Figure 1: Effects of delays on the dynamical properties of the iMLS circuit. A) The activation response time T_{ON}, normalized by the response time of the sTF T_0, is plotted as a function of the repression level p_{ss}/p_0 for different values of the delay in miRNA activation (see legend on the right) and for an illustrative case of a delay in the mRNA dynamics (dashed blu line). The parameter values are the same of Figure 2 of the main text with $\tau_s/\tau_r = 1$. The curve corresponding to the circuit dynamics in absence of delay (red continuous line) is reported for reference. B) Using the same parameter values, the response time T_{OFF}, normalized by the response time of the sTF T_0 , is shown for different delay values as a function of the repression level. C) The dynamical response of the host gene protein concentration to a two step input is depicted for different delay values (see the legend in the B plot). The two-step input function and the parameter values are the same of Figure C in the main text, in particular of the plot i in the bottom right corner. The plot shows that the peaks in the protein concentration response become progressively higher increasing the delay in miRNA activation. The opposite is true if there is a time-lag between transcription and mRNA translation (blue curve). D) The sensitivity S, normalized with $2CV_p$ (where $CV_p = \sigma_p/\langle p \rangle$ is the noise level at steady state), is reported as a function of the miRNA activation delay time for the parameter values of the previous plot C. In absence of delay the circuit is not sensitive (as indeed shown also in Figure 4C of the main text), but a slow miRNA activation leads to an output signal above the noise level before adaptation.

the maturation steps or an inefficient mRNA splicing leads to a mRNA level in the cytoplasm lower than the transcribed one. While in our model each transcription event generates an active miRNA and a target mRNA, the effect of different effective synthesis rates for the two molecular species should not alter qualitatively the results presented in this paper if the final levels of the active molecular species are proportional to the transcribed ones. In fact, in this case eventual discrepancies between the transcribed level and the active level of miRNAs or mRNAs could actually be taken into account rescaling the appropriate parameters. For example, the fact that only a certain proportion of the transcribed miRNAs becomes active is phenomenologically equivalent to a rescaling of the repression strength $1/h$, since the relevant quantity that establishes the dowregulation level of target translation is s/h.

Finally, there is experimental evidence of competition for a finite RISC pool (or other necessary small RNA processing or transport machinery) in transfection experiments [12] (although the relevance of this competition is still quantitatively less clear for endogenous miRNAs). If the RISC concentration is limiting, a saturation effect is expected for high miRNA concentrations since the available RISC complexes can be not enough to ensure activation of all the present miRNAs. In this case, an increase of transcription does not lead to a proportional increase of the active miRNA concentration and thus to an increased level of repression. Therefore, for a low RISC concentration compared to the miRNA one, the functions of miRNA- mediated circuits can be compromised, as it has been shown for the adaptive behaviour of incoherent feedforward loops [11]. Consequently, the relative amount of RISC complexes (as well as other processing enzymes) and miRNAs is a factor potentially relevant in possible experimental tests of the results presented in this paper.

6 Bioinformatic analysis - Supplementary tables

A)

Total Ensembl Gene Identifiers (ENSG)	22257
Total Ensembl Transcript Identifiers (ENST)	22257
Total exons (ENSE)	216139
Total miRNA gene names uniqued	676

B)

Total of Intergenic_miRNAs uniqued	325
Total of Intragenic miRNAs uniqued	351

	Intragenic miRNA	Intronic	Exonic	UTR
miRNA	351	331	20	7
Host_Genes	318	296	22	7
miRNA::Host genes	372	350	22	7
miRNA_Same_Strand	302	288	15	6
Host genes_Same_Strand	273	258	15	6
miRNA::Host genes_SS	312	297	15	6
miRNA_Opposite_Strand	57	52	5	1
Host genes_Opposite_Strand	48	41	7	1
miRNA::Host_genes_OS	60	53	7	1

Figure 2: Table 1S. (A) Description of human known protein coding genes and human miRNA datasets. For each ENSG, we considered the longest Ensembl transcript ID (ENST). Data from Ensembl v.57 that include miRBase v.13. (B) Classification of human miRNAs with respect to their host genes. Depending on the genomic location of human miRNAs, we considered as intergenic miRNAs whose genomic position were found distant from annotated genes, while intragenic miRNAs whose located within a transcript (annotated as "host gene"). Afterward, intragenic miRNAs were further subdivided into intronic and exonic. An intragenic miRNA was called exonic if its genomic coordinates overlap with genomic coordinates of any exon in the database, and was labeled intronic otherwise. In addition, intragenic miRNAs can be classified depending on whether they are on the same strand (SS) or on the opposite strand (OS) of their host gene. All UTR miRNAs were found to overlap the exon regions.

References

1. Rosenfeld N, Elowitz MB and Alon U (2002) Negative autoregulation speeds the response times of transcription networks. *J Mol Biol* **323:** 785-793.

2. Bintu L, Buchler NE, Garcia HG, Gerland U, Hwa T, Kondev J, Phillips R (2005) Transcriptional regulation by the numbers: models. *Curr Opin Genet De* **15(2):** 116-24.

3. Goentoro L, Shoval O, Kirschner MW, Alon U (2009) The incoherent feedforward loop can provide fold-change detection in gene regulation. *Mol Cell* **36:** 894-899.

4. Osella M, Bosia C, Corá D and Caselle M (2011) The role of incoherent microRNA mediated feedforward loops in noise buffering. *PLos Comput Biol*, **7(3):** e1001101.

5. Komorowski M, Miekisz J and Kierzek AM (2009) Translational repression contributes grater noise to gene expression than transcriptional repression. *Biophys J* **96(2):** 372-84.

6. Thattai M and van Oudenaarden A (2001) Intrinsic noise in gene regulatory networks. *Proc Natl Acad Sci USA* **98:** 8614-8619.

7. van Kampen N G, Stochastic processes in physics and chemistry, first edition 1981, Elsevier Science & Technology Books.

8. Levine E, Kuhlman T, Zhang Z, Hwa T (2007) Quantitative characteristics of gene regulation by small RNA. *PLoS Biology* **9:** 229.

9. Mukherji S, Ebert M S, Zheng G X Y, Ysang J S, Sharp P A and van Oudenaarden A (2011) MicroRNAs can generate thresholds in target gene expression. *Nature Genetics* **43(9):** 854-860.

10. Kim YK, Kim VN (2007) Processing of intronic microRNAs. *EMBO J* **26(3):**775–783,

11. Bleris L, Xie Z, Glass D, Adadey A, Sontag E, Benenson Y (2011) Synthetic incoherent feedforward circuits show adaptation to the amount of their genetic template. *Mol Syst Biol* **7:** 519.

12. Khan AA, Betel D, Miller ML, Sander C, Leslie CS, Marks DS. (2009) Transfection of small RNAs globally perturbs gene regulation by endogenous microRNAs. *Nat Biotechnol* **27(6):** 549-55.

13. Tsang J, Zhu J and van Oudenaarden A (2007) MicroRNA-mediated feedback and feedforward loops are recurrent network motifs in mammals. *Mol Cell*, **26:** 753-767.

14. Megraw M, Sethupathy P, Gumireddy K, Jensen S T, Huang Q and Hatzigeorgiou A G (2009) Isoform specific gene auto-regulation via miRNAs: a case study on miR-128b and ARPP-21. *Theor Chem Acc*, **125:** 593-598.

iMSLs	TargetScan	miRanda	DIANA microT	PITA	PicTar	RNA22		MirTarget2	TargetMiner	Total prediction Algorithm
						3'UTR	5'UTR			
hsa-miR-196a-1|HOXB7|ENSG00000120087	x			x	x			x	x	5
hsa-miR-330|EML2|ENSG00000125746	x	x	x	x				x		5
hsa-miR-608|SEMA4G|ENSG00000095539		x	x	x				x	x	5
hsa-miR-196b|HOXA9|ENSG00000078399	x			x				x	x	4
hsa-miR-224|GABRE|ENSG00000102287	x		x	x		x				4
hsa-miR-10a|HOXB3|ENSG00000120093	x			x				x		3
hsa-miR-128-2|ARPP-21|ENSG00000172995 (*)				x	x				x	3
hsa-miR-26-2|CTDSP2|ENSG00000175215	x				x				x	3
hsa-miR-28|LPP|ENSG00000145012		x	x						x	3
hsa-miR-103-1|PANK3|ENSG00000120137						x			x	2
hsa-miR-126|EGFL7|ENSG00000172889		x	x							2
hsa-miR-338|AATK|ENSG00000181409	x		x							2
hsa-miR-361|CHM|ENSG00000188419			x						x	2
hsa-miR-489|CALCR|ENSG00000004948			x						x	2
hsa-miR-502|CLCN5|ENSG00000171365			x	x						2
hsa-miR-505|ATP11C|ENSG00000101974				x				x		2
hsa-miR-532|CLCN5|ENSG00000171365			x						x	2
hsa-miR-548k|PPFIA1|ENSG00000131626				x				x		2
hsa-miR-548n|DPY19L1|ENSG00000173852				x				x		2
hsa-miR-555|ASH1L|ENSG00000116539			x						x	2
hsa-miR-561|GULP1|ENSG00000144366			x					x		2
hsa-miR-582|PDE4D|ENSG00000113448			x	x						2
hsa-miR-597|TNKS|ENSG00000173273			x					x		2
hsa-miR-615|HOXC5|ENSG00000172789		x	x							2
hsa-miR-885|ATP2B2|ENSG00000157087		x	x							2
hsa-let-7f-2|HUWE1|ENSG00000086758		x					x			2
hsa-let-7g|WDR82|ENSG00000164091							x			1
hsa-miR-107|PANK1|ENSG00000152782			x							1
hsa-miR-125b-2|C21orf34|ENSG00000215386	x									1
hsa-miR-1255a|PPP3CA|ENSG00000138814				x						1
hsa-miR-1271|ARL10|ENSG00000175414	x									1
hsa-miR-149|GPC1|ENSG00000063660			x							1
hsa-miR-153-1|PTPRN|ENSG00000054356		x								1
hsa-miR-185|C22orf25|ENSG00000183597			x							1
hsa-miR-186|ZRANB2|ENSG00000132485			x							1
hsa-miR-188|CLCN5|ENSG00000171365			x							1
hsa-miR-24-1|C9orf3|ENSG00000148120		x								1
hsa-miR-32|C9orf5|ENSG00000106771			x							1
hsa-miR-335|MEST|ENSG00000106484			x							1
hsa-miR-346|GRID1|ENSG00000182771			x							1
hsa-miR-362|CLCN5|ENSG00000171365			x							1

Figure 3: Table 2S. List of intronic miRNA-mediated self loops (iMSLs). For each iMSL we highlighted with "x" the correspondent dataset where they were found, with blue color if they were reported by [13], with red if they were found by [14] and with fuchsia if they were reported in both these two studies. The only iMSL experimentally validated was highlighted with (∗) symbol.

iMSLs	TargetScan	miRanda	DIANA microT	PITA	PicTar	RNA22		MirTarget2	TargetMiner	Total prediction Algorithm
						3'UTR	5'UTR			
hsa-miR-448|HTR2C|ENSG00000147246		x								1
hsa-miR-483|IGF2|ENSG00000129965			x							1
hsa-miR-483|INS|ENSG00000167244			x							1
hsa-miR-488|ASTN1|ENSG00000152092			x							1
hsa-miR-500|CLCN5|ENSG00000171365			x							1
hsa-miR-501|CLCN5|ENSG00000171365			x							1
hsa-miR-511-1|MRC1|ENSG00000120586			x							1
hsa-miR-511-2|MRC1L1|ENSG00000183748			x							1
hsa-miR-548d-1|ATAD2|ENSG00000156802								x		1
hsa-miR-548f-5|DMD|ENSG00000198947				x						1
hsa-miR-548f-2|ERBB4|ENSG00000178568				x						1
hsa-miR-548f-1|PCDH15|ENSG00000150275				x						1
hsa-miR-548e|SHOC2|ENSG00000108061									x	1
hsa-miR-548p|ST8SIA4|ENSG00000113532								x		1
hsa-miR-579|ZFR|ENSG00000056097			x							1
hsa-miR-581|ARL15|ENSG00000185305			x							1
hsa-miR-603|KIAA1217|ENSG00000120549			x							1
hsa-miR-605|PRKG1|ENSG00000185532			x							1
hsa-miR-609|C10orf79|ENSG00000197748		x								1
hsa-miR-613|APOLD1|ENSG00000178878			x							1
hsa-miR-623|PHGDHL1|ENSG00000134882			x							1
hsa-miR-624|STRN3|ENSG00000196792		x								1
hsa-miR-626|AC073657.2|ENSG00000174197			x							1
hsa-miR-627|VPS39|ENSG00000166887			x							1
hsa-miR-628|CCPG1|ENSG00000214882			x							1
hsa-miR-637|DAPK3|ENSG00000167657		x								1
hsa-miR-640|GATAD2A|ENSG00000167491			x							1
hsa-miR-641|AKT2|ENSG00000105221			x							1
hsa-miR-644|ITCH|ENSG00000078747									x	1
hsa-miR-657|AATK|ENSG00000181409			x							1
hsa-miR-660|CLCN5|ENSG00000171365			x							1
hsa-miR-661|PLEC1|ENSG00000178209			x							1
hsa-miR-708|ODZ4|ENSG00000149256			x							1
hsa-miR-765|ARHGEF11|ENSG00000132694			x							1
hsa-miR-766|SEPT6|ENSG00000125354		x								1
hsa-miR-9-1|C1orf61|ENSG00000125462							x			1
**Total iMSLs predicted (x each algorithm)**	9	14	51	16	3	2	3	13	13	

Figure 4: Sequel of Table 2S.
